# Supplementary material for: Exploring genotype by environment interaction on cassava yield and yield related traits using classical statistical methods
Source: PLoS One. 2022 Jul 18;17(7):e0268189. doi: 10.1371/journal.pone.0268189 (PMC9292083; doi:10.1371/journal.pone.0268189)
Supplement: S1 Fig — (PDF) [file pone.0268189.s001.pdf]

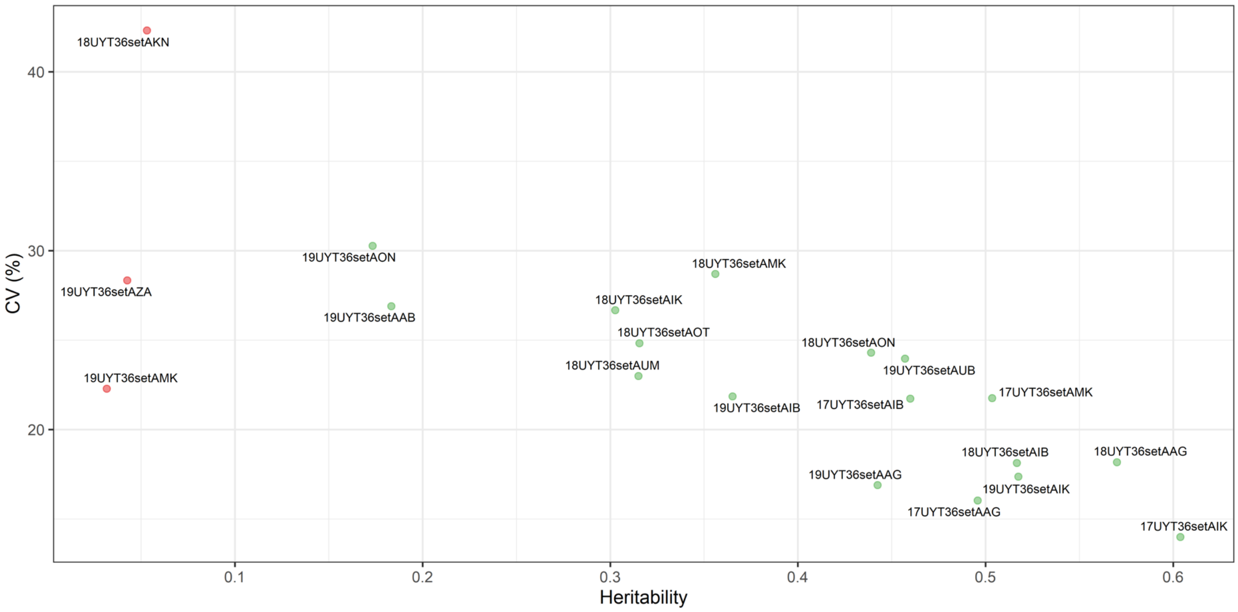

S1 Fig. Scatter plot of coefficient of correlation (CV %) versus heritability assessing data quality of individual trials for fresh root yield (t/ha). Trials whose CV above 40.5 or heritability below 0.05 displayed in a red data point were removed from combined analysis.
