# Supplementary figures and images for: Exploring genotype by environment interaction on cassava yield and yield related traits using classical statistical methods
Source: PLoS One. 2022 Jul 18;17(7):e0268189. doi: 10.1371/journal.pone.0268189 (PMC9292083; doi:10.1371/journal.pone.0268189)

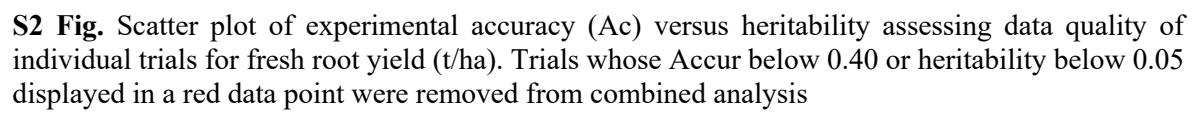

Supplement: S2 Fig — (PDF) [file pone.0268189.s002.pdf]
