# Supplementary material for: Exploring genotype by environment interaction on cassava yield and yield related traits using classical statistical methods
Source: PLoS One. 2022 Jul 18;17(7):e0268189. doi: 10.1371/journal.pone.0268189 (PMC9292083; doi:10.1371/journal.pone.0268189)
Supplement: S3 Fig — (PDF) [file pone.0268189.s003.pdf]

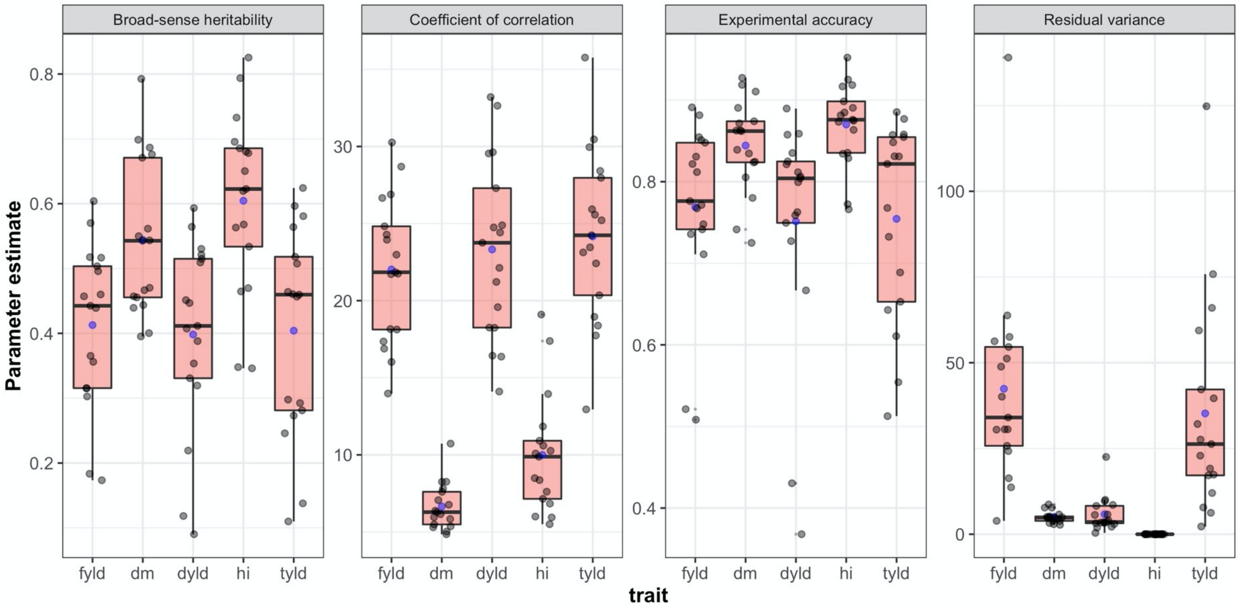

S3 Fig. Boxplot showing distribution of fresh root yield (FYLD t/ha), dry matter content (DMC %), dry yield (DYLD t/ha), harvest index (HI), and top yield (TYLD t/ha) for derived parameters such as broad-sense heritability, coefficient of variation, experimental accuracy, and residual variance across 17 trials.
