# Supplementary material for: Exploring genotype by environment interaction on cassava yield and yield related traits using classical statistical methods
Source: PLoS One. 2022 Jul 18;17(7):e0268189. doi: 10.1371/journal.pone.0268189 (PMC9292083; doi:10.1371/journal.pone.0268189)
Supplement: S5 Fig — (PDF) [file pone.0268189.s005.pdf]

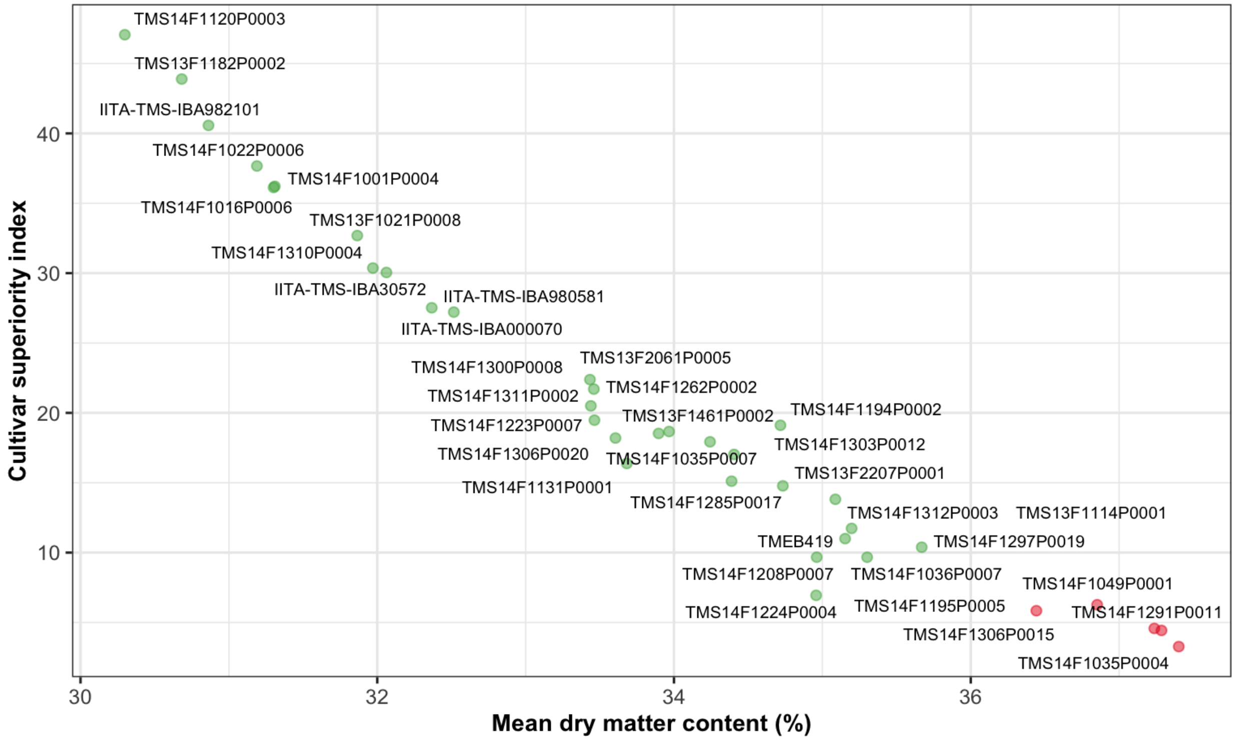

S5 Fig. Scatter plot showing cultivar superiority index versus mean dry matter content of 36 cassava clonal lines. Clonal lines in red data points are high yielding and most stable across the testing environments
