# Supplementary material for: Exploring genotype by environment interaction on cassava yield and yield related traits using classical statistical methods
Source: PLoS One. 2022 Jul 18;17(7):e0268189. doi: 10.1371/journal.pone.0268189 (PMC9292083; doi:10.1371/journal.pone.0268189)
Supplement: S7 Fig — (PDF) [file pone.0268189.s007.pdf]

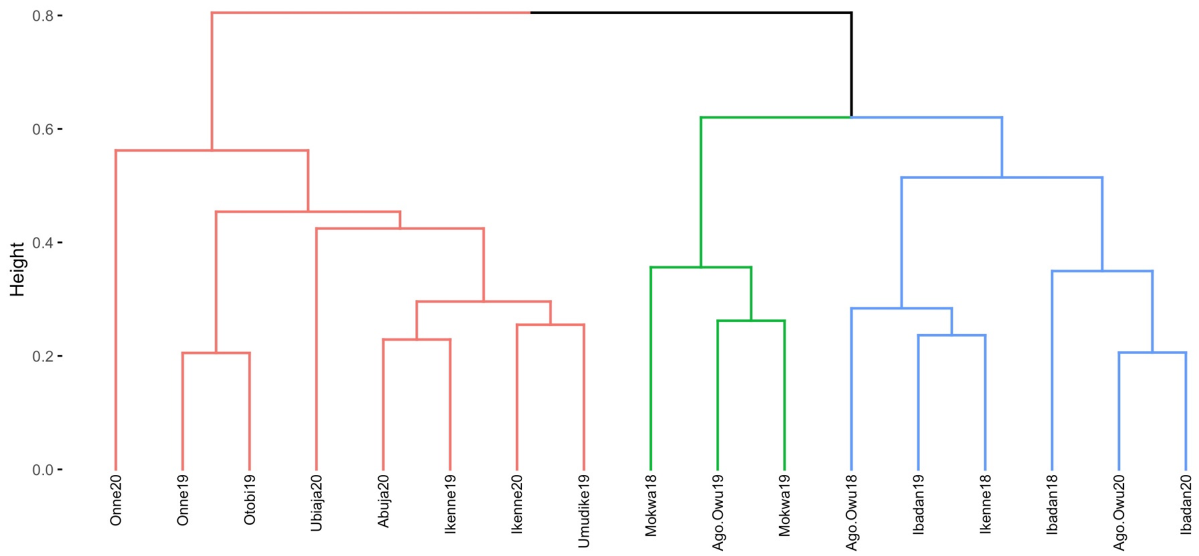

S7 Fig. A cluster dendogram showing relatedness among the testing environments based on distance matrix derived from correlation among environments genotypic BLUPs of fresh root yield (t/ha). The clustering was based on ward.D2 linkage method.
