# Supplementary material for: Exploring genotype by environment interaction on cassava yield and yield related traits using classical statistical methods
Source: PLoS One. 2022 Jul 18;17(7):e0268189. doi: 10.1371/journal.pone.0268189 (PMC9292083; doi:10.1371/journal.pone.0268189)
Supplement: S8 Fig — (PDF) [file pone.0268189.s008.pdf]

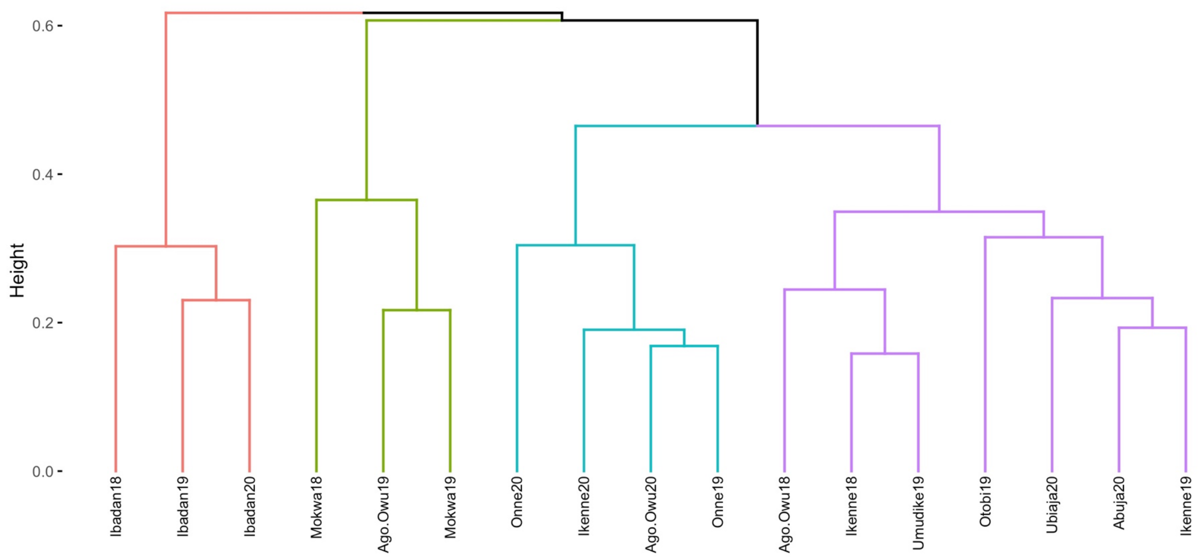

S8 Fig. A cluster dendogram showing relatedness among the testing environments based on distance matrix derived from correlation among environments genotypic BLUPs of dry matter content. The clustering was based on ward.D2 linkage method.
