# Supplementary material for: Exploring genotype by environment interaction on cassava yield and yield related traits using classical statistical methods
Source: PLoS One. 2022 Jul 18;17(7):e0268189. doi: 10.1371/journal.pone.0268189 (PMC9292083; doi:10.1371/journal.pone.0268189)
Supplement: Attachment — Submitted filename: PONE-D-21-40030.pdf [file pone.0268189.s027.pdf]

PLOS ONE

Exploring genotype by environment interaction on cassava yield and yield related traits using classical statistical methods

--Manuscript Draft--

**Manuscript Number:**	PONE-D-21-40030
**Article Type:**	Research Article
**Full Title:**	Exploring genotype by environment interaction on cassava yield and yield related traits using classical statistical methods
**Short Title:**	Accounting for genotype by environment interaction in cassava improvement
**Corresponding Author:**	Jean-Luc Jannink<br>USDA-ARS / Cornell University<br>Ithaca, UNITED STATES
**Keywords:**	genotype by environment interaction (GEI); target population of environment (TPE); linear-bilinear models; genotype sensitivity; yield; heritability; best linear unbiased predictor (BLUP)
**Abstract:**	Variety advancement decisions for root quality and yield-related traits in cassava are complex due to the variable genotype patterns by environment interactions (GEI). Therefore, studies focused on the dissection of the existing patterns of GEI using linear-bilinear models such as Finlay-Wilkinson (FW), additive main effect and multiplicative interaction (AMMI), and genotype and genotype by environment interaction (GGE) models are critical in defining the target population of environments (TPEs) for future testing, selection, and advancement. This study assessed thirty-six elite cassava clones in nine locations over three cropping seasons in the cassava breeding program of IITA based in Nigeria with a view of quantifying the GEI effects for root quality and yield-related traits. Genetic correlations coefficients and heritability estimates among environments depicted mostly intermediate to high values indicating high correlations with the major TPE. There was a differential clonal rankings among the environments indicating existence of GEI as also revealed by likelihood ratio test (LRT) which further confirmed statistical model with heterogeneity of error variances across the environments fit better. For all fitted models, we found the main effects of environment, genotype and their interaction to be significant for all observed traits except for dry matter content whose GEI sensitivities was marginally significant as reported in FW. We identified TMS14F1297P0019 and TMEB419 as two topmost stable genotypes with sensitivities value of 0.63 and 0.66 respectively using FW model. However, GGE and The AMMI stability value in conjunction with genotype selection index revealed that IITA-TMS-IBA000070 and TMS14F1036P0007 are the top-ranking genotypes combining both stability and yield performance measures. AMMI-2 model clustered the testing environments into 6 mega-environment based on winning genotypes for fresh root yield. Alternatively, we identified 3 clusters of testing environments based on genotypic blups derived from random GEI component.
**Order of Authors:**	Moshood Bakare
	Siraj Kayondo
	Cynthia Aghogho
	Marnin Wolfe
	Elizabeth Parkes
	Peter Kulakow
	Chiedozie Egesi
	Ismail Rabbi
	Jean-Luc Jannink
**Additional Information:**	
**Question**	**Response**

Powered by Editorial Manager® and ProduXion Manager® from Aries Systems Corporation

**Financial Disclosure**<br><br>Enter a financial disclosure statement that describes the sources of funding for the work included in this submission. Review the submission guidelines for detailed requirements. View published research articles from *PLOS ONE* for specific examples.<br><br>This statement is required for submission and **will appear in the published article** if the submission is accepted. Please make sure it is accurate.<br><br>**Unfunded studies**<br>Enter: *The author(s) received no specific funding for this work.*<br><br>**Funded studies**<br>Enter a statement with the following details:<br>• Initials of the authors who received each award<br>• Grant numbers awarded to each author<br>• The full name of each funder<br>• URL of each funder website<br>• Did the sponsors or funders play any role in the study design, data collection and analysis, decision to publish, or preparation of the manuscript?<br>• **NO** - Include this sentence at the end of your statement: *The funders had no role in study design, data collection and analysis, decision to publish, or preparation of the manuscript.*<br>• **YES** - Specify the role(s) played.<br><br>* typeset	This research was generously funded by the Bill & Melinda Gates Foundation (https://www.gatesfoundation.org) and the United Kingdom's Foreign, Commonwealth & Development Office (FCDO) (https://www.gov.uk/government/organisations/foreign-commonwealth-development-office) through the "Next Generation Cassava Breeding project" (https://www.nextgencassava.org) (Grant INV-007637) managed by Cornell University.<br>Copyright: ©2021 Bakare et al. This is an open access research article distributed and licensed under the Creative Commons Attribution License (CC-BY), that permits unrestricted use, distribution, and reproduction in any medium, provided the original author and source are credited.<br>The funders gave support in the implementation of this study and encourage in publishing this work.
**Competing Interests**<br><br>Use the instructions below to enter a competing interest statement for this submission. On behalf of all authors, disclose any competing interests that could be perceived to bias this work—acknowledging all financial support and any other relevant financial or non-financial competing interests.<br><br>This statement is required for submission	The authors have declared that no competing interests exist..

Powered by Editorial Manager® and ProduXion Manager® from Aries Systems Corporation

and **will appear in the published article** if the submission is accepted. Please make sure it is accurate and that any funding sources listed in your Funding Information later in the submission form are also declared in your Financial Disclosure statement.<br><br>View published research articles from *PLOS ONE* for specific examples.<br><br>**NO authors have competing interests**<br><br>Enter: *The authors have declared that no competing interests exist.*<br><br>**Authors with competing interests**<br><br>Enter competing interest details beginning with this statement:<br><br>*I have read the journal's policy and the authors of this manuscript have the following competing interests: [insert competing interests here]*<br><br>* typeset	
**Ethics Statement**<br><br>Enter an ethics statement for this submission. This statement is required if the study involved:<br><br>• Human participants<br>• Human specimens or tissue<br>• Vertebrate animals or cephalopods<br>• Vertebrate embryos or tissues<br>• Field research<br><br>Write "N/A" if the submission does not require an ethics statement.<br><br>General guidance is provided below. Consult the submission guidelines for detailed instructions. **Make sure that all information entered here is included in the Methods section of the manuscript.**	N/A

**Format for specific study types**<br><br>**Human Subject Research (involving human participants and/or tissue)**<br>• Give the name of the institutional review board or ethics committee that approved the study<br>• Include the approval number and/or a statement indicating approval of this research<br>• Indicate the form of consent obtained (written/oral) or the reason that consent was not obtained (e.g. the data were analyzed anonymously)<br><br>**Animal Research (involving vertebrate animals, embryos or tissues)**<br>• Provide the name of the Institutional Animal Care and Use Committee (IACUC) or other relevant ethics board that reviewed the study protocol, and indicate whether they approved this research or granted a formal waiver of ethical approval<br>• Include an approval number if one was obtained<br>• If the study involved *non-human primates*, add *additional details* about animal welfare and steps taken to ameliorate suffering<br>• If anesthesia, euthanasia, or any kind of animal sacrifice is part of the study, include briefly which substances and/or methods were applied<br><br>**Field Research**<br><br>Include the following details if this study involves the collection of plant, animal, or other materials from a natural setting:<br>• Field permit number<br>• Name of the institution or relevant body that granted permission	
**Data Availability**<br><br>Authors are required to make all data underlying the findings described fully available, without restriction, and from the time of publication. PLOS allows rare exceptions to address legal and ethical concerns. See the PLOS Data Policy and FAQ for detailed information.	Yes - all data are fully available without restriction

A Data Availability Statement describing where the data can be found is required at submission. Your answers to this question constitute the Data Availability Statement and **will be published in the article**, if accepted.<br><br>**Important:** Stating 'data available on request from the author' is not sufficient. If your data are only available upon request, select 'No' for the first question and explain your exceptional situation in the text box.<br><br>Do the authors confirm that all data underlying the findings described in their manuscript are fully available without restriction?	
**Describe where the data may be found in full sentences. If you are copying our sample text, replace any instances of XXX with the appropriate details.**<br><br>• If the data are **held or will be held in a public repository**, include URLs, accession numbers or DOIs. If this information will only be available after acceptance, indicate this by ticking the box below. For example: *All XXX files are available from the XXX database (accession number(s) XXX, XXX.).*<br>• If the data are all contained **within the manuscript and/or Supporting Information files**, enter the following: *All relevant data are within the manuscript and its Supporting Information files.*<br>• If neither of these applies but you are able to provide **details of access elsewhere**, with or without limitations, please do so. For example:<br><br>*Data cannot be shared publicly because of [XXX]. Data are available from the XXX Institutional Data Access / Ethics Committee (contact via XXX) for researchers who meet the criteria for access to confidential data.*<br><br>*The data underlying the results presented in the study are available from (include the name of the third party*	All data files and analysis scripts are available online on github url address https://github.com/mab658/classical_analysis_GxE

and contact information or URL).<br>• This text is appropriate if the data are owned by a third party and authors do not have permission to share the data.<br><br>* typeset	
Additional data availability information:	

Manuscript

Click here to access/download;Manuscript;Manuscript classical analysis of GxE.docx

Exploring genotype by environment interaction on cassava yield and yield related traits using classical statistical methods

Moshood A. Bakare[1*], Siraj Ismail Kayondo[2], Cynthia I. Aghogho[2,3], Marnin D. Wolfe[1], Elizabeth Y. Parkes[2], Peter Kulakow[2], Chiedozie Egesi[1,2,4], Ismail Yusuf Rabbi[2], and Jean-Luc Jannink[1*]

[1] Plant Breeding and Genetics Section, School of Integrative Plant Science, College of Agriculture and Life Sciences, Cornell University, 14853, Ithaca, NY USA.
[2] International Institute of Tropical Agriculture (IITA), PMB 5320, Oyo Road, Ibadan, Nigeria.
[3] West Africa Centre for Crop Improvement, University of Ghana, PMB LG 30, Legon, Ghana
[4] National Root Crops Research Institute Umudike, Umuahia, Nigeria

*Corresponding authors
E-mail: jeanluc.work@gmail.com (JJ)

Abstract

Variety advancement decisions for root quality and yield-related traits in cassava are complex due to the variable genotype patterns by environment interactions (GEI). Therefore, studies focused on the dissection of the existing patterns of GEI using linear-bilinear models such as Finlay-Wilkinson (FW), additive main effect and multiplicative interaction (AMMI), and genotype and genotype by environment interaction (GGE) models are critical in defining the target population of environments (TPEs) for future testing, selection, and advancement. This study assessed thirty-six elite cassava clones in nine locations over three cropping seasons in the cassava breeding program of IITA based in Nigeria with a view of quantifying the GEI effects for root quality and yield-related traits. Genetic correlations coefficients and heritability estimates among environments depicted mostly intermediate to high values indicating high correlations with the major TPE. There was a differential clonal rankings among the environments indicating existence of GEI as also revealed by likelihood ratio test (LRT) which further confirmed statistical model with heterogeneity of error variances across the environments fit better. For all fitted models, we found the main effects of environment, genotype and their interaction to be significant for all observed traits except for dry matter content whose GEI sensitivities was marginally significant as reported in FW. We identified TMS14F1297P0019 and TMEB419 as two topmost stable genotypes with sensitivities value of 0.63 and 0.66 respectively using FW model. However, GGE and The AMMI stability value in conjunction with genotype selection index revealed that IITA-

TMS-IBA000070 and TMS14F1036P0007 are the top-ranking genotypes combining both stability and yield performance measures. AMMI-2 model clustered the testing environments into 6 mega-environment based on winning genotypes for fresh root yield. Alternatively, we identified 3 clusters of testing environments based on genotypic blups derived from random GEI component.

Introduction

Cassava (*Manihot esculenta Crant*[illegible] one of the most important food crops worldwide, particularly in sub-Saharan Africa [1,2]. It is known to be a significant source of carbohydrates in the diet of millions of people in developing countries. It is cultivated under diverse edaphic and climatic conditions throughout the world [3] due to its efficient carbohydrate production [4] among staple root crops. Cassava is a food security crop grown predominantly by smallholders for subsistence due to its adaptability to survive in drought-prone areas under marginal conditions where other crops may not thrive [1,5]. In comparison to other crops, Sayre et al. [5] reported that cassava is mostly grown under marginal conditions, making it produce more energy per unit area with limited human input than other crops. Cassava is getting much attention because of its mechanisms to cope with diverse environmental conditions [6]. Cassava shows a strong genotype by environment interaction (GEI) effects [7], which makes selection for superior genotypes a difficult task for cassava breeders. Therefore, selection for a superior genotype requires the cassava breeding program to take into consideration the GEI effect. Detailed evaluation of the magnitude and significance of GEI is of utmost importance to ensure greater precision in the release of high yielding and stable genotypes [7].

Crop phenotypes are well known to be influenced by environmental conditions [8]. This can result in differential genotypic responses across the testing environments resulting in GEI variability. The phenotypic panel for evaluating GEI is often called a multi-environment trial (MET). In METs where genetic lines are often evaluated over many years and locations within a target population of environments (TPE), there is usually an important cross-over interaction (COI), and a GEI term needs to be explored to study the non-additivity of effects.

Among several statistical models devised for exploring the empirical genotypic mean response across environments and for studying and interpreting GEI in agricultural field trials are:

Linear models, bilinear models, and linear-bilinear models [9]. Typical examples of fixed-effect linear-bilinear models such as the Sites Regression (SREG) [10] and the Additive Main Effect and Multiplicative Interaction (AMMI) models [11,12] are used for investigating patterns of genotypic response across environments. In these models, biplots can be used to visualize the patterns of genotypic responses and environments [13,14] that allow the breeders to identify high or low performing genotype(s) with broad or specific adaptation for a given trait of interest. A form of the fixed-effect linear model called a factorial regression (FR) model, and a form of the bilinear model, called partial least squares (PLS) regression, allow integrating external environmental and genotypic covariates into the model and can be used to identify weather conditions causing GEI or the genomic segments (e.g., molecular markers) influencing GEI [9].

AMMI is one of the commonly used fixed-effect linear-bilinear models that models the complex structure of GEI. It is a hybrid statistical model combining analysis of variance (ANOVA) to model main effects of genotype and environment and principal component analysis (PCA) to decompose complex GEI structure into Interactive Principal Component Axes (IPCAs) through singular value decomposition. In this model, the percentage of GEI variation explained by IPCAs decreases, with the first IPCA accounting for the highest percentage of GEI variation. The AMMI biplot of first IPCA scores against the mean of genotypic performance visualizes both genotypes and environments through which genotypes with broad or specific adaptation can be identified. Genotypes with IPCA score in the vicinity of zero are considered to be stable across environments. However, genotypes with scores that deviate from zero for a given IPCA are unstable relative to the determinants of that IPCA but may exhibit specific adaptation if they are identified as close to a particular environment in the AMMI biplot. AMMI is often preferable to a linear regression approach in the sense of being parsimonious as it requires fewer degrees of freedom to explain

GEI. The AMMI model can be further used to delineate the testing environments with the best genotypes into mega environment using principal component axes scores and AMMI stability values (ASV) [15]. The AMMI stability value (ASV) for a genotype is defined to be the distance from the coordinate point for that genotype to the origin in a two-dimensional space of the first two Interactive principal component analysis scores (IPCA1 and IPCA2, [16]. Because IPCA scores account for different amounts of variation in the GEI sum of squares a weighted value must be assigned in the assessment of stability using the AMMI model. Genotypic stability alone does not provide a sufficient yardstick for selection as stable genotypes might not necessarily give the highest yield performance. Mahmodi et al. [17] and Tumuhimbise et al. [2] used a genotype selection index (GSI) which is sum of genotypic yield rank across environments and ASV rank to identify high yielding and stable genotypes. This index implicitly values yield and stability equally. A low GSI value signifies a desirable genotype with high average yield performance and high stability [17].

The Site Regression (SREG) model, also called Genotype Main Effect plus Genotype-Environment Interaction (GGE), is a modification of AMMI model where the bilinear term combines the genotype main effect (G) and the GEI effect in a multiplicative term. It allows breeders to explore total genetic rather than exclusively GEI variation. GGE allows the finding of GEI in terms of crossover resulting from changes in genotypic ranking across the environments [18]. Unlike AMMI biplots that approximate only GEI, the genotypic scores in a GGE model describe the G and GEI jointly to approximate overall performance of (G + GE) interaction.

This study's principal objectives were: (i) To identify stable and high-yielding cassava clones adapted to broad and/or specific environments; (ii) To determine the relative importance of sources of variation influencing key agronomic traits; and (iii) To identify mega environments for

Nigerian cassava; iv) To provide a clear road map for other researchers pursuing these types of objective.

Materials and methods

Clonal material and experimental field design

Thirty-six (36) advanced cassava clonal lines were evaluated, corresponding to 31 experimental lines and five standard checks (Table 1) as in the uniform yield trial (UYT), an advanced stage of the IITA breeding program. These clones were evaluated across 20 trials grown in nine (9) locations across different agro-ecological zones in Nigeria from 2017 to 2020 (Table 2). The geographical coordinate system describing the location of each field trial in space is given in terms of latitude and longitude. Weather data were collected across the testing environments from an online database, www.awhere.com. Mean temperature over crop growth cycle across the environments ranged from 26.8 °C for Zaria20 to 28.9 °C for Mokwa20. Meanwhile, the total precipitation varied between 944.2 for Kano19 and 3208.4 for Onne19. Average relative humidity varied across Each trial was established as a Randomized Complete Block Design (RCBD) in three replicates. The experimental plot consisted of 6 rows of length 5.6 m with an inter-row spacing of 1 m and intra-row spacing of 0.8 m. The locations used varied from one cropping season to another as did the number of trials, resulting in an unbalanced data structure.

Table 1. Thirty-six cassava clonal lines evaluated across 20 environments in Nigeria

Clone	Pedigree
IITA-TMS-IBA000070	
IITA-TMS-IBA30572	58308 X BRANCA DE SANTA CATARINA
IITA-TMS-IBA980581	
IITA-TMS-IBA982101	IITA-TMS-IBA951181

TMEB419	
TMS13F1021P0008	IITA-TMS-IBA010903 X IITA-TMS-IBA030075
TMS13F1114P0001	IITA-TMS-IBA070126 X IITA-TMS-IBA000355
TMS13F1182P0002	IITA-TMS-IBA011412 X TMEB419
TMS13F1461P0002	IITA-TMS-MM990268 X IITA-TMS-IBA000355
TMS13F2061P0005	(IITA-TMS-IBA070004 X IITA-TMS-IBA070520 X SM3361-30)-11
TMS13F2207P0001	IITA-TMS-KAN930061 X IITA-TMS-IBA960249
TMS14F1001P0004	TMS13F1303P0001 X TMS13F1020P0002
TMS14F1016P0006	TMS13F1307P0011 X TMS13F1108P0007
TMS14F1022P0006	TMS13F1307P0020 X TMS13F1106P0006
TMS14F1035P0004	TMS13F1095P0009 X TMS13F1307P0008
TMS14F1035P0007	TMS13F1095P0009 X TMS13F1307P0008
TMS14F1036P0007	TMS13F1109P0009 X TMS13F1307P0020
TMS14F1049P0001	TMS13F1391P0039 X TMS13F1306P0003
TMS14F1120P0003	TMS13F1309P0001 X TMS13F`1333P0003
TMS14F1131P0001	TMS13F1087P0002 X TMS13F1176P0003
TMS14F1194P0002	TMS13F1101P0007 X TMS13F1307P0020
TMS14F1195P0005	TMS13F1106P0006 X TMS13F1307P0020
TMS14F1208P0007	TMS13F1106P0006 X TMS13F1020P0002
TMS14F1223P0007	TMS13F1106P0006 X TMS13F1108P0007
TMS14F1224P0004	TMS13F1106P0006 X TMS13F1212P0055
TMS14F1262P0002	TMS13F1063P0009 X TMS13F1307P0008
TMS14F1285P0017	IITA-TMS-IBA961632 X IITA-TMS-IBA000070
TMS14F1291P0011	IITA-TMS-IBA030055A X IITA-TMS-IBA961632
TMS14F1297P0019	IITA-TMS-IBA020431 X IITA-TMS-MM970806
TMS14F1300P0008	IITA-TMS-ZAR930151 X IITA-TMS-MM970043
TMS14F1303P0012	I IITA-TMS-ZAR930151 X ITA-TMS-IBA930134
TMS14F1306P0015	IITA-TMS-IBA030060 X IITA-TMS-MM970043
TMS14F1306P0020	IITA-TMS-IBA030060 X IITA-TMS-MM970043
TMS14F1310P0004	IITA-TMS-IBA030060 X IITA-TMS-IBA930265
TMS14F1311P0020.	IITA-TMS-IBA030060 X IITA-TMS-ZAR930151
TMS14F1312P0003	IITA-TMS-IBA930134 X IITA-TMS-ZAR930151

128

Table 2. Climatic characteristics of the experimental sites showing the geographical coordinates, range and mean of temperature, precipitation, and relative humidity.

**Climatic factors**	**Abuja**	**Ago-owu**	**Ibadan**	**Ikenne**	**Kano**	**Mokwa**	**Onne**	**Otobi**	**Ubiaja**	**Umudike**	**Zaria**
Latitude (°)	9.67 N	7.25 N	7.37 N	6.87 N	12.00 N	9.28 N	4.72 N	7.10 N	6.64 N	5.48 N	11.09 N
Longitude (°)	7.39 E	4.32 E	3.94 E	3.71 E	8.59 E	5.05 E	7.15 E	8.08 E	6.39 E	7.54 E	7.71 E
**Min - Max (Mean) temperature (°C)**											
2018	-	23.9 - 32.6 (28.3)	23.6 - 32.2 (27.9)	24.3 - 32.1 (28.2)	-	23.6 - 33.9 (28.8)	-	-	-	-	-
2019	-	23.9 - 32.1 (28.0)	23.7 - 32.2 (27.9)	24.4 - 32.2 (28.3)	20.9 - 34.1 (27.5)	23.7 - 33.9 (28.8)	21.8 - 29.5 (25.7)	23.1 - 32.2 (27.7)	.	23.1 - 30.9 (27.0)	-
2020	22.8 - 32.9 (27.8)	23.7 - 32.3 (28.0)	23.5 - 32.2 (27.8)	24.3 - 32.4 (28.3)	-	23.5 - 34.3 (28.9)	21.9 - 29.7 (25.8)	-	22.6 - 31.5 (27.0)	-	20.5 - 33.2 (26.8)
**Total precipitation (mm)**											
2018	-	1082.1	1175.9	1295.4	-	1215.7	-	-	-	-	-
2019	-	1647.4	1439.0	1653.2	944.2	1250.5	3208.4	1409.1	-	2795.6	-
2020	1920.0	1673.8	1479.1	1681.3	-	1068.5	3199.9	.	2050.0		1322.8
**Relative humidity (%)**											
2018	-	56.9 - 88.5 (72.7)	57.5 - 88.4 (72.9)	61.6 - 91.0 (76.3)	-	46.3 - 77.9 (62.1)	-	-	-	-	-
2019	-	59.4 - 90.9 (75.2)	58.2 - 90.1 (74.2)	62.3 - 92.5 (77.4)	31.2 - 68.3 (49.8)	45.4 - 78.9 (62.1)	62.8 - 95.0 (78.9)	53.8 - 86.8 (70.3)	-	62.1 - 93.5 (77.8)	-

2020	51.8 - 86.0 (68.9)	58.0 - 91.7 (74.9)	56.4 - 90.6 (73.5)	60.3 - 93.1 (76.7)	-	44.8 - 79.4 (62.1)	61.8 - 95.6 (78.7)	-	56.7 - 91.5 (74.1)	-	33.7 - 71.6 (52.6)
Agroecological Zone	Southern Guinea savanna	Derived savanna	Forest savanna transition	Humid forest	Northern Guinea savanna	Southern Guinea savanna	Humid forest	Southern Guinea savanna	Humid forest	Humid forest	Northern Guinea savanna

Statistical analysis

Data quality control and single trial analysis

Before formal genotype by environment analysis, the empirical distribution of the observed agronomic traits was visualized by individual environment (i.e., location by year combination) in boxplots using the ggplot2 package [19] in R [20]. A linear mixed model was fitted to the individual trials to estimate clonal variance components and broad-sense heritability. The Proc Mixed procedure of Statistical Analysis Software (SAS) software version 9.4 [21] was used to fit the following model:

$$y = \mu + X_1 r + X_2 cov + Z_1 g + \epsilon \qquad (1)$$

where y is the vector (n × 1) of observed phenotypic values, in which n is the number of observations; μ is the intercept (overall mean); r is the vector (j × 1) of fixed effects of j replicates; *cov* denotes the proportion of plant stands harvested, a covariate for all traits except dry matter content; g is the vector (i × 1) of random effects of ith genotype with its associated design matrix Z_1, and $\in$ is a residual term which is assumed to follow Gaussian distribution.

Quality of each trial was assessed by calculating the coefficient of variation (CV), broad-sense heritability (H^2), and experimental accuracy (Ac) proposed by [22] using the following expressions: $CV\% = \frac{\hat{\sigma}_e}{\bar{y}} \times 100$, $H^2 = \frac{{\hat{\sigma}_g}^2}{{\hat{\sigma}_g}^2 + {\hat{\sigma}_e}^2}$, and $Ac = \sqrt{1 - \frac{\overline{PEV}}{{\hat{\sigma}_g}^2}}$

in which $\hat{\sigma}_e$ is the estimated residual standard deviation, $\bar{y}$ is the estimated overall mean for a trait; σ^2_g is the estimated genetic variance σ^2_e is the estimated error variance, and $\overline{PEV}$ is the average of prediction error variance.

This analysis identified three trials with low heritability ($H^2 < 0.1$), low accuracy (Ac < 0.4) and high CV (CV > 40.5). These trials were removed from further analysis (Figs 2 and 3).

Joint G×E analysis of multiple trials

Following single trial analysis to identify and eliminate poor quality trials, we carried out a combined analysis of trials using a linear mixed model framework on data consisting of *g* genotypes evaluated across *e* environment in *r* replicates within each environment. The model fitted for each agronomic trait was:

$$y_{ijk} = \mu + g_i + e_j + b_{k(j)} + ge_{ij} + cov + \epsilon_{ijk} \quad (2)$$

where y_{ijk} is a phenotypic vector of the observed agronomic trait of ith genotype in k^{th} replicate within j^{th} environment; μ is a fixed intercept, g_i is the effect of ith genotype considered to be random, e_j is the random effect of jth environment, $b_{k(j)}$ is the random block effect within j^{th} environment, ge_{ij} is the random interaction effect of ith genotype and j^{th} environment, *cov* denotes the proportion of plant stands harvested as covariates; and $\in_{ijk}$ is the vector of the random residual term. The random effects in the model are postulated to follow a multivariate normal distribution with means and variances defined as:

$g \sim N(0, \sigma^2_g I)$, $e \sim N(0, \sigma^2_e I)$, $b \sim N(0, \sigma^2_b I)$, $ge \sim N(0, \sigma^2_{ge} I)$, and $\in \sim N(0, \sigma^2_{\in} I)$

where 0 is the expected value (mean) of zero; σ^2_g is the genetic variance; σ^2_e is the environmental variance; σ^2_b is the block variance nested with j^{th} environment; σ^2_{ge} is the variance of genotype-by-environment interaction $\sigma^2_{\in}$ is the residual variance; and *I* is the identity matrix, with order equal to the number of observations. We calculated the percentage of total phenotypic variance explained by each random effect to determine how significant it was in influencing the variability in each trait. Broad-sense heritability on plot mean basis across all environments was derived from variance components estimate as

$$H^2 = \frac{\sigma_g^2}{\sigma_g^2 + \left(\sigma_{ge}^2/e\right) + \left(\sigma_\epsilon^2/er\right)} \quad (3)$$

where *e* is the number of environment, *r* is the number of replicates of genotypes per environment, and other terms were described above. Out of 17 trials, 12 had replicates of 3 and others have 2 replicates each. Therefore, the harmonic mean was calculated to be approximately 2.6 and it was used as a good representative of the number of replicates across all the trials while computing the heritability.

We further ascertained the presence or absence of GEI by fitting both the reduced model without the GEI term and a full model that included the GEI term. The likelihood ratio test (LRT) was carried out on each of the agronomic traits to determine if there was a significant improvement in fitting a full model. In the same manner, we tested for the homogeneity versus heterogeneity of error variance across trials. Finally, we further partitioned the GEI variance into a repeatable component as genotype by location (GL), and non-repeatable components as genotype by year (GY) and genotype by location by year (GLY). In the presence of significant GEI, we assessed its pattern by fitting Finlay Wilkinson (FW), Additive main effect and multiplicative interaction model (AMMI), and genotype and genotype by environment (GGE) models on the two-way genotype environment adjusted means using the statgenGxE package [23] in R [20], as described below.

Finlay-Wilkinson regression

The Finlay-Wilkinson regression approach [24] was used to model GEI by regressing mean phenotypic performance of individual genotypes on an environmental index and determine the heterogeneity of associated slopes. The environmental index was the mean of all clones for a trait in an environment. Without explicit environmental information, an environment can be

characterized by the mean performance of all genotypes in that environment [25]. This method requires two steps: (i) Compute the environmental mean, and (ii) Estimate intercept and slope for each genotype by regressing genotypic performance on the environmental index. Prior to fitting the Finlay Wilkinson model, trait values were scaled to mean of zero and standard deviation of one following the equation below to allow comparison of means square error (MSE) values across traits, which are measured in different scale of units as:

$$y_{ij_{standardized}} = \left[y_{ij} - mean(Y)\right]/sd(Y) \qquad (4)$$

where y_{ij} is the adjusted phenotypic mean value of ith genotype in j^{th} environment and Y is the overall mean of adjusted phenotypic response of all clones in all environments. This standardization of each trait necessitated that the MSE values reflected variability and not the absolute scale of a given unit [26]. Then for each trait, we fitted the Finlay-Wilkinson model as

$$y_{ij} = \mu + g_i + \beta_i e_j + \epsilon_{ij} \qquad (5)$$

in which y_{ij} is as described above but scaled, μ is overall mean, g_i is the genotypic intercept, β_i is a slope representing the sensitivity of ith genotype. The average value of β_i is 1; $\beta_i > 1$ indicates genotypes with a higher than average sensitivity, and $\beta_i < 1$ indicates genotypes that are less sensitive than average, e_j is the environment sample mean, and $\in_{ij}$ is a random error term associated with i^{th} genotype evaluated in j^{th} environment.

The three key parameters arising from this approach are: the genotypic intercept, which expresses the general performance of a genotype, the slope which measures the sensitivity of a genotype, and the residual variance which is a deviation from the regression line denoting the stability. The residual is genotypic-specific. Therefore, some lines have high residual, while others are low. To quantify and compare trait sensitivity to GEI, the variance of slope and that of MSE resulting from Finlay-Wilkinson model were used [26]

AMMI analysis

The observed traits' G×E interaction was analyzed using the Additive Main effect and Multiplicative interaction (AMMI) model. AMMI is a fixed effect linear-bilinear model which analyses the main effect of genotype and environment using ANOVA and the multiplicative effect using principal component analysis (PCA) in a single model [27]. Each of the agronomic traits was subjected to AMMI analysis by fitting the model

$$y_{ij} = \mu + g_i + e_j + \sum_{k=1}^{K} \left(\lambda_k \alpha_{ik} \gamma_{jk}\right) + \epsilon_{ij} \qquad (6)$$

where y_{ij} is the mean performance of ith genotype in j^{th} environment; μ is the intercept; g_i is fixed effect of ith genotype; e_j is the fixed effect of jth environment. The GEI component is decomposed into K multiplicative terms (k = 1, 2, …, K), each multiplicative term is a product of kth eigenvalue (λ_k); genotypic score (α_{ik}); and environmental scores (γ_{jk}); and $\in_{ij}$ is the residual GEI not captured by the model and some error deviation.

We computed AMMI Stability Value (ASV) for each genotype relative to the influence of IPCA1 and IPCA2 scores based on their interaction sum of squares according to Purchase [16] using the formula:

$$ASV = \sqrt{\left[\left(\frac{SS_{IPCA1}}{SS_{IPCA2}}\right) \times IPCA1\right]^2 + IPCA2^2} \qquad (7)$$

where (SS_{IPCA1}/SS_{IPCA2}) was the weight assigned to the *IPCA1* value by dividing the *IPCA1* SS by the *IPCA2* SS; and the *IPCA1* and *IPCA2* scores were the genotypic score derived from the AMMI model. A large positive ASV value indicates a genotype that is more adapted to particular environments. A small (close to zero) ASV value indicates a stable genotype across environments [16].

We also calculated genotype selection index (GSI) for each genotype as the sum of genotypic rank based on mean yield across environments (RY) and rank of AMMI stability value (RASV):

$$GSI_i = RASV_i + RY_i \tag{8}$$

The genotype with the lowest GSI value is considered the most valuable [28].

GGE analysis

Genotype main effect and Genotype by Environment (GGE) analysis is a modification of AMMI analysis. Unlike AMMI, only the environment is fitted as a main effect in the GGE model. This brings about fitting principal component analysis jointly on genotype main effect and genotype by environment interaction as a sum of multiplicative terms. The GGE analysis does the job of fitting the principal component model with two components to the two-way genotype by environment table of mean centered per environment with genotypes as object and environments as variable [18]. Like AMMI, the principal component scores can be exploited in constructing biplots. The GGE model or AMMI analysis could be used at differentiating mega-environment [29] even though this is debated in the literature in terms of preference. The observed traits were subjected to GGE analysis by fitting GGE model as

$$y_{ij} = \mu + e_j + \sum_{k=1}^{2} \left(\lambda_k \alpha_{ik} \gamma_{jk}\right) + \epsilon_{ij} \tag{9}$$

where each term is similar to the AMMI model.

Mega-environment delineation

In the context of GEI, a mega-environment is defined to be a group of environments sharing a common best performing genotype. In principle, it also follows that different genotypes are adapted to different mega-environments and GEI variation between the mega-environments is

higher than variation within the mega-environments [30]. We determined mega-environments based on the AMMI-2 model of order 2. The environments were clustered using the *gxeMegaEnv()* function of the statgenGxE package [23] based on the fitted values from AMMI-2 model. Environments that share the common best genotype belong to the same mega-environment.

The version of GGE biplot graphic called "which won where" plot is also a tool for the delineation of a mega-environment. In the case of delineating mega-environment through GGE biplot analysis, the resulting mean value in the graphics is related to mega-environment mean and not grand mean, and it supports in identifying genotypes with broad or narrow adaptation to some environments or groups of environments [31]. The "which won where" biplot includes an irregular polygon whose vertices mark the genotypes that are furthest from the origin in all directions such that the polygon encompasses all genotypes in the biplot. Lines are also drawn originating from the biplot's origin and intercepting the polygon's sides perpendicularly [32]. The lines originating from the origin split the biplot into sections and the genotype at the vertex of every section had the optimal yield performance in environments contained in that section. Each section in effect defines a mega-environment.

Cultivar superiority index

Further assessment of stability of each clone was determined after testing the significance of GEI. We quantified yield stability across the testing environments using a univariate stability estimate called cultivar-superiority measure [33]. It is a measure of stability by superiority index and it is defined as a function of the sum of the squared differences between a cultivar's mean performance and the best cultivar's mean, where the sum is across trials. Lin and Binns [33] proposed the calculation of superiority index using expression:

$$P_i = \sum_{j=1}^{n} \left(X_{ij} - M_j\right)^2 / 2n \quad (10)$$

where P_i is the superiority index of ith cultivar; X_{ij} is the yield of ith cultivar in the jth environment; M_j is the highest yield response got among the cultivars in the jth environment; and *n* is the number of environments. This expression was further decomposed as

$$P_i = \left[n(\bar{X}_{i.} - \bar{M})^2 + \sum_{j=1}^{n} \left(X_{ij} - M_j + \bar{M}\right)^2 \right] \Big/ 2n \quad (11)$$

where $\bar{X}_{i.} = \sum^{n}_{j=1} X_{ij}/n$, and $\bar{M} = \sum^{n}_{j=1} M_j/n$, $\bar{X}_{i.}$ = mean yield of ith cultivar in *n* environments and $\bar{M}$ = mean of maximum response in the *n* environments. According to Lin and Binns [33], the first section of P_i expression quantifies genetic deviation and the second section signifies GEI. Cultivars with the lowest values of the index P_i incline to be more stable, and in proximity to the best cultivar in each environment.

Representative of target population of environments

We considered all environments in the study to be the target population of environments (TPE). We identified testing environments that best represented the TPE by following these steps: i) calculate environment-specific genotypic BLUPs by fitting genotype effect as random, ii) calculate genotypic BLUPs across all environments which represent a TPE, iii) calculate the Pearson correlation between environment-specific genotypic BLUPs and genotypic BLUPs across all environments as a measure of breeding value accuracy, and iv) estimate environment-specific heritability based on the Cullis approach which involves the variance of a difference between genotypes. Cullis et al. [34] proposed to compute heritability as

$$H^2_{Cullis} = 1 - \frac{\bar{V}^{BLUP}_{\Delta..}}{2\sigma^2_g} \tag{12}$$

where $\bar{v}_{\Delta..}^{BLUP}$ is the mean variance of a difference of two genotypic BLUPs and σ^2_g is the genetic variance, and v) rank heritability and Pearson correlation value and take the sum of their rank. We use both high genetic correlation and heritability estimate as indicators for identifying a good representative for the TPE.

To determine the number of testing environments representing the entire TPE, we randomly sampled subsets of 1 to 16 environments from the phenotypic data repeatedly for 50 times. For each sampling, a model was fitted to obtain genotypic best linear unbiased prediction (BLUP). Then, for each sampling environment, Pearson correlation was obtained between the BLUP and the BLUP derived from all the environments. We further calculated the average correlation coefficient as a breeding value accuracy relative to overall environments. The point at which the line plot showing the trends of breeding values accuracy relative to all TPE and sampled environments reaches a plateau is used to determine optimal number of environments to represent TPE.

To provide further insights into the relatedness or grouping of the current testing environments based on key traits, we extracted environment-specific genotypic BLUPs from the random G×E effect component of the joint analysis. Then, we further carried out a Pearson correlation analysis among the environments. Thereafter, the clustering of the environments was carried out based on a distance matrix derived from correlation matrix using ward.D2 linkage method [35]. Intuitively, we examined the resulting dendogram from the clustering to identify environments that joined together with the smallest distance as a cluster group

Results

Phenotypic data description and single trial analysis

The distribution of the phenotypic values of observed traits of the 36 genotypes revealed that all observed traits approximated a normal distribution across the testing environments satisfying the assumption of normality in classical statistical methods (Fig 1). We observed a range in variation of fresh root yield (t/ha) from low performance environments (Onne19, Onne20, Ubiaja20), to high performance environments (Ibadan19, Otobi19, Ago-owu20, Ikenne20). The boxplots further revealed the heterogeneity of variability for the observed traits across the environments indicating the presence of GEI.

Fig 1. Box plot displaying the distribution of dry matter content (dm %), dry yield (dyld t/ha), fresh root yield (fyld t/ha), harvest index (hi), and top yield (tyld t/ha) of 36 genotypes evaluated.

The plots resulting from data quality control of single-trial analysis (S1 and S2 Figs.) showed that the three trials: 18UYT36setAKN, 19UYT36setAZA, and 19UYT36setAMK should be removed based on thresholds set for CV, H^2, and Accr. The mean fresh root yield across the remaining 17 trials ranged from 6.52 t/ha (19UYT36setAON) in Onne20 to 47.49 t/ha (18UYT36setAOT) in Otobi19 with an overall mean of 27.46 t/ha (S1 Table). The summary statistics for other traits like dry matter content, dry yield, top yield, and harvest index from each testing environment are also reported (S1 Table). In addition, visualization of the distribution of derived parameters such as broad-sense heritability, coefficient of variation, experimental accuracy, and residual variance for all traits across all trials were showed (S3 Fig). We observed smallest variability in dry matter content and then harvest index across all trials relative to other traits.

Combined analysis of multiple trials

The likelihood ratio test (LRT) statistics identified presence of significant GEI and error variance heterogeneity across testing environments for all observed traits (S2 and S3 Tables). The percentage of phenotypic variance attributed to each model term for each trait was reported (Fig 2 and S4 Table). The environment had a significant effect on all traits ($P < 0.01$) and captured largest percentage of total phenotypic variance ranging from 48.6% in harvest index to 63.9% in top yield. The genotypic effect was highly significant ($P < 0.001$) for each trait and explained percentage of phenotypic variance between 2.6% (harvest index) and 12.6% (dry matter content). The GEI term was also highly significant ($P < 0.001$) and accounted for 5.3% (top yield) to 12.5% (harvest index) of phenotypic variance. We observed relatively high GEI variance compared to genetic variance for fresh root yield. In contrast, genetic variance for top yield was higher than GEI variance indicating environmental conditions played a lesser role in influencing top yield (Fig 2 and S4 Table). The replication nested within environment captured between 2.3-5.6% of the phenotypic variance, which was the smallest relative to other source of variation. It was highly significant ($P < 0.01$) for all traits. Residual term was the second largest source of variation after the environment effect. It accounted between 18.8-30.8% of the phenotypic variance (Fig 2 and S4 Table). The broad-sense heritability estimates varied from 0.64 for harvest index to 0.92 for dry matter content (S4 Table).

Fig 2 Barchart plot displaying the percentage of total phenotypic variance attributed to each effect for each trait across 17 trials.

Further decomposition of GEI term into repeatable component (GL) and non-repeatable component (GY and GLY) revealed that GY component was not significant for all traits except dry matter content ($P < 0.001$). It accounted for the smallest portion of phenotypic variance ranging from approximately 0.2 to 5.6% (S5 Table). Repeatable GL component explained between 3.7-

48.6% percentage of phenotypic variance. In comparison to GL and GY components, the GLY term was highly significant (P < 0.001) and accounted for largest portion of phenotypic variance for all traits except for harvest index where GL explained largest portion of phenotypic variance. The significance of GLY is an indication that for all traits, genotypic response to conditions particular to a specific location depends on year of evaluation and vice versa..

Finlay-Wilkinson regression

The genotypic and environmental main effects of the Finlay-Wilkinson (FW) model were highly significant (P <= 0.001) for all observed traits (S6 Table). Significant differences in regression slope (sensitivity) among genotypes on the environmental mean was found for all traits except dry matter content (S6 Table). In other words, there was variation in genotypic response for all traits but not dry matter with respect to changes in environment mean.

The genotypic sensitivity values were ranked from the most stable (low sensitivity value) to the least stable (high sensitivity value) for each trait (S7 Table). The FW model identified TMS14F1297P0019, TMEB419, TMS14F1120P0003, TMS13F1461P0002, and TMS14F1312P0003 as the top 5 most stable genotypes for fresh root yield with sensitivities values of 0.638, 0.663, 0.721, 0.786, and 0.813 respectively.

Trait sensitivity to GEI was quantified by the variance of the slopes and the variance of MSEs. The top yield had the lowest median MSE of all traits (median = 0.146) and the variance of MSE (variance=0.007) (Fig 3 and S8 Table). Meanwhile, the slope variance varied from 0.023 (top yield) to 0.058 (harvest index) with the corresponding slope median values of 1.001 and 1.010 respectively, (Fig 3 and S9 Table).

Fig 3. Boxplots showing distribution of MSE and slope resulting from Finlay Wilkinson model for the evaluation of 36 elites cassava clones across 17 environments for 5 traits.

AMMI analysis

The AMMI analysis revealed significant variation in the main effects of genotype (G), environment (E) and their interactions (GEI) ($P < 0.001$) for all observed traits (S10 Table). The partition of total sum of squares (TSS) showed that the environment main effect accounted for highest amount of variation varying from 48.2% (harvest index) to 76.1% (top yield).

The decomposition of variation in GEI for fresh root yield showed that the first and the second interactive principal components (IPCs) captured 21.6% and 15.7% and accounted for 4.5% and 3.3% of the TSS. For dry matter content, the first two IPCs accounted for 21.0% and 15.9% of GEI SS and 4.3% and 3.3% of TSS. Finally, the partition of variation in GEI for top yield revealed the first and second principal components explained 26.5% and 17.6% and accounted for 4.0% and 2.7% of TSS respectively (S10 Table).

The AMMI-2 biplot revealed how the genotypes and environment are interrelated based on fresh root yield (Fig 4a). The genotypes close to each other in this biplot have a tendency to have a relatively similar yield in the tested environments. Meanwhile, genotypes far apart within the plot tend to differ in yield or have a unique pattern of yield response across the environments. Genotypes in the vicinity of the origin are not sensitive to environmental interaction and those distant from the origin are sensitive and have large interaction.

Fig 4. Polygon view of (a) AMMI2 model and (b) GGE2 model for fresh root yield (t/ha).

The mean fresh root yield (t/ha) value of cassava genotypes averaged over testing environments indicated that genotype IITA-TMS-IBA000070 had the highest fresh root yield (37.9 t/ha) and genotype TMS14F1120P0003 had the lowest yield (22.5 t/ha, S11 Table). The IPC1 and IPC2 scores signify the adaptability of a genotype over environments and the relationship between genotype and environment (S11 Table). Genotypes with large scores in absolute value

(e.g., TMS14F1297P0019 and TMEB419) have high interactions and are unstable, whereas genotypes with scores close to zero (IITA-TMS-IBA000070, TMS14F1036P0007) have low interactions and are stable.

The AMMI stability value (ASV) ranged from 2.60 to 40.34, averaging 15.39 across the 36 cassava genotypes. The genotypes IITA-TMS-IBA000070 (2.60), TMS14F1306P0020 (3.28), TMS14F1223P0007 (3.88), and TMS14F1306P0015 (5.68) had the lowest ASV values, while TMEB419 (40.34), TMS14F1297P0019 (39.21), and TMS14F1300P0008 (30.41) had the highest values (S11 Table). Stability is not the only yardstick for selection, as the most stable genotype would not necessarily give the best yield performance. Therefore, the genotype selection index showed that IITA-TMS-IBA000070 and TMS14F1036P0007 were the two best when combining both stability and yield performance measures (S11 Table).

Identifying mega environments

The fitted fresh root yield values from the AMMI2 model were used to cluster the seventeen testing environments into six mega environments, one for each of the winning genotypes "IITA-TMS-IBA000070", "IITA-TMS-IBA980581", "TMS14F1016P0006", "TMS14F1036P0007", "TMS14F1285P0017", and "TMS14F1300P0008' (S12 Table). The genotypes IITA-TMS-IBA000070 and TMS14F1016P0006 had broad adaptation to eight and four environments, respectively. However, genotypes IITA-TMS-IBA980581, TMS14F1285P0017, and TMS14F1300P0008 had specific adaptation to environments Abuja20, Mokwa18, and Ibadan18, respectively. TMS14F1036P0007 was the best genotype in environments Ago-Owu19 and Ibadan20.

GGE analysis

The GGE model showed a significant main effect of environment and combined genotype and genotype by environment interaction effect (P <= 0.001) for the observed traits (S13 Table). The partition of TSS which includes sum of squares (SS) of environment and genotype and genotype by environment interaction indicated that environment explained a larger percentage of variation for all observed traits relative to GGE component except for harvest index. The variation explained by the GGE component ranged from 23.9% (top yield) to 51.8% (harvest index). For fresh root yield, the first and second IPCs accounted for 9.6% and 4.4% of TSS and explained 33.3% and 15.3% of GGE variation, respectively with a cumulative total of 48.6%. For dry matter content, the first two IPCs captured 17.4% and 4.3% of TSS and explained 47.6% and 11.8% of GGE variation, with a cumulative total of 59.4%. For the top yield, the first two IPCs explained 11.3% and 3.1% of TSS and captured 47.4% and 12.8% of GGE variation resulting to cumulative total of 60.2%.

GGE biplots based on symmetric scaling of genotype and environment were used to estimate the pattern of environments in relation to genotypes (Fig 4b). The first principal component of environment had both negative and positive scores indicating a difference in yield performance across environments resulting in cross-over GEI.

Fig 4. Polygon view of (a) AMMI2 model and (b) GGE2 model for fresh root yield (t/ha).

The three models revealed that environment effect accounted for almost the same percentage of total phenotypic variation for the observed traits (Fig 5). Likewise, the genotypic effect of FW and AMMI models explained nearly the same percentage of total phenotypic variation for each measurable trait. The interaction factor of GGE model includes main effect of genotype and genotype by environment interact resulting in a larger percentage of total phenotypic variation in

comparison to other models. The GEI component of AMMI captured larger percentage of variation than FW, which explained relatively low variation for all the traits. For all the observed traits, the residual term of the AMMI model was the lowest while for FW it was the highest (Fig 5).

Fig 5. Percentage of total variation captured by each factor from fitting additive main effect and multiplicative interaction (AMMI), Finlay Wilkinson (FW), and genotype and genotype by environment (GGE) models to yield related traits on 36 elite cassava genotypes evaluated in 17 environments. Note that the variation attributed to genotype x environment factor for GGE model includes that of genotype.

Cultivar superiority index

Mean performance and index values of cultivar-superiority stability estimates were presented for fresh root yield, dry matter content, and dry yield to assess genotypes' stability across the testing environments (S14 Table). Among the 36 cassava genotypes, 19 had mean fresh root yield above grand mean of 29.5 t/ha. The remaining genotypes had average fresh root yield below grand mean. Dyke et al. [36] pointed out that a stable genotype tends to sustain a constant yield performance across testing environments. Consequently, genotypes with above mean performance and are stable by outcome of this stability measures are desirable.

Superiority index value P_i is defined as the deviation of the ith genotype relative to the genotype with maximum performance in each environment. The top ranked 5 stable genotypes for fresh root yield that tends to be closer to the best genotype in each environment were identified with lowest P_i value include IITA-TMS-IBA000070, TMS14F1036P0007, TMS14F1016P0006, TMS14F1262P0002, and TMS14F1035P0004, most of which would be attributed to genetic deviation [33]. These genotypes also have relatively high fresh root yield above grand average yield of 29.5 t/ha and their corresponding dry matter ranged from 31.3% for TMS14F1016P0006 to 37.4% for TMS14F1035P0004 (S4 Fig and S14 Table). Top ranked 5 genotype showing

consistent performance for dry matter content include TMS14F1035P0004, TMS14F1306P0015, TMS14F1291P0011, TMS14F1195P0005, and TMS14F1049P0001 (S5 Fig and S14 Table). However, it is superiority index associated to dry yield that showed genotypes with consistent performance for both fresh root yield and dry matter content and top ranked 5 genotypes were TMS14F1036P0007, IITA-TMS-IBA000070, TMS14F1035P0004, TMS14F1262P0002, and TMS13F2207P0001 (S6 Fig and S14 Table).

Representative of target population of environments

The correlation coefficient of each environment's BLUPs with genotypic BLUPs of all environments in the TPE for fresh root yield ranged from 0.33 (Ibadan18) to 0.73 (Ago-Owu19) with corresponding heritability estimates of 0.71 and 0.80, respectively (Table 3). The top ranked 5 environments showing high correlation with TPE and high heritability estimate include Ago-Owu19 (0.73, 0.80), Ikenne18 (0.69, 0.82), Ibadan19 (0.68, 0.73), Ago-Owu18 (0.56, 0.74), and Ikenne20 (0.67,0.68) (Table 3).

Table 3. Correlation coefficient (r) of environment_specific BLUPs with all target population of environment (TPE) and environment-specific heritability (H^2) based on the Cullis method [34] for fresh root yield (t/ha).

**Environment**	**r**	**(H^2)**	**rank(r)**	**rank(H^2)**	**Sum ranks**
Ago-Owu19	0.73	0.80	1	2	3
Ikenne18	0.69	0.82	2	1	3
Ibadan19	0.68	0.73	4	5	9
Ago-Owu18	0.56	0.74	9	4	13
Ikenne20	0.67	0.68	5	8	13
Onne19	0.67	0.69	6	7	13
Ago-Owu20	0.68	0.61	3	11	14
Mokwa18	0.47	0.74	12	3	15

Ibadan20	0.61	0.51	7	15	22
Umudike19	0.61	0.56	8	14	22
Ibadan18	0.33	0.71	17	6	23
Ikenne19	0.54	0.57	10	13	23
Mokwa19	0.46	0.61	13	10	23
Ubiaja20	0.42	0.62	15	9	24
Otobi19	0.44	0.58	14	12	26
Abuja20	0.49	0.27	11	16	27
Onne20	0.40	0.26	16	17	33

As for dry matter content, the environments revealed a higher range of correlation coefficient with TPE relative to fresh root yield varying from Onne20 (0.48) to Ikenne20 (0.85) with corresponding heritability estimate of 0.57 and 0.88, respectively (S15 Table). The top ranked 5 environments to represent the TPE for showing high correlation and high heritability included Ikenne20 (0.85, 0.88), Ikenne18 (0.79, 0.78), Onne19 (0.72, 0.87), Ubiaja20 (0.73, 0.82), and Umudike (0.78, 0.77). For top yiel[illegible]ere was higher variability in the correlation coefficient with TPE ranging from 0.14 (Mokwa19) to 0.83 (Ubiaja20) with heritability estimates of 0.32 and 0.71, respectively (S16 Table). The top ranked 5 environments to represent the TPE were Ikenne19 (0.82, 0.81), Otobi19 (0.83, 0.78), Ikenne18 (0.79, 0.82), Ago-Owu20 (0.79, 0.76), and Ubiaja20 (0.83, 0.71). A line graph provides further insights into the number of environment(s) that is likely to be sampled to represent TPE and their corresponding breeding value accuracy compared to all the environment for fresh root yield, dry matter content, and top yield (Fig 6). Regardless of the number of environments sampled, the breeding value accuracy of fresh root yield is lower compared to dry matter content and top yield. As revealed in Fig 6, sampling of five (5) environments is likely to represent TPE where fresh root yield has an approximate breeding value

accuracy of 0.84 lower than dry matter content and top yield with breeding value accuracy of 0.92 and 0.91 respectively.

Fig 6. A line graph showing the estimated breeding value accuracy against the number of sampling environments for dry matter content (dmc), fresh root yield (fyld), and top yield (tyld).

The relatedness among the testing TPE for fresh root yield revealed the grouping of the testing TPE into three cluster groups such that environments within a cluster are more similar and dissimilar from environments in other cluster (S7 Fig). As for dry matter content, the TPEs were grouped into 4 clusters (S8 Fig). However, 6 cluster groups of TPE were identified for top yield out of which 3 clusters have one environment each (S9 Fig).

Discussion

This study demonstrated the application of classical ANOVA in a linear mixed model framework and linear-bilinear models such as Finlay-Wilkinson, additive main effect and multiplicative interaction model, and genotype plus genotype environment models towards identifying stable genotypes, mega-environments and environments representative of the TPE.

The large sum of squares and significant effect of environment on the observed agronomic traits as shown by FW, AMMI, and GGE models demonstrated that the field trials were conducted under diverse environmental conditions causing variation in cassava genotypes yield and other yield-related traits. The significant variation of GEI effect found for the observed agronomic traits indicated that neither genotype nor environment effect can independently capture all the variation observed. This resulted in diverse performance of the genotypes in the testing environments. This variation is useful when proposing to examine GEI, as well as to assess the stability of genotypes. We found that the AMMI model attributed largest percentage of treatment sum of squares to environment (71.1%) for fresh root yield. This finding was contrary to Tumuhimbise et al. [2] who reported that genotype accounted for largest percentage of treatment sum of squares (48.5%). The disparity in the result may be due to the fact that our study evaluated 36 genotypes in 17 environments compared to 12 genotypes in 3 environments in [illegible]umuhimbise et al. [2]. Also contrary to our findings, Jiwuba et al. [37] reported that GEI accounted for largest percentage of treatment sum of squares (43.80%) in their study where 60 genotypes were evaluated over 6 environments.

As for top yield, the environment captured largest percentage of total variability (76.1%) from the AMMI model. This was contrary to findings from Jiwuba et al. [37] who reported that environment accounted for the lowest percentage of total variation (11.9%) for the biomass. The

disparity in the result may be because they evaluated fewer environments (6) in their study compared to 17 environments in our study. Unlike fresh root yield and top yield, the percentage of total sum of squares attributed to G (16.1%) was relatively close to GEI (20.6%) for dry matter content, indicating that there was similarity in dry matter response of some of the genotypes across environments. However, all the linear bilinear models explored in this study revealed that the environment accounted for much greater variation on DMC compared to genotypic effect. This may be due to the fact this is a UYT study, so that genotypes have already been strongly selected, so that genetic variability is reduced. In contrast, Benesi et al. [38] reported that genotypic influence on dry matter content is much higher than for the environment.

AMMI, like the FW model, revealed a significant genotypic effect for the observed agronomic traits, signifying the presence of genetic variation in IITA cassava germplasm. This is similar to what Nduwumuremyi et al. [39] reported about the existence of significant genetic variation in Rwandan germplasm.

The limitation of classical ANOVA is that it does not provide insight into the complex pattern of GEI, which necessitates further use of linear bilinear models. The strength of AMMI and GGE models is that they concurrently visualize genotypes and environments using biplots that expedite the interpretation of GEI. On biplots, a genotype in the vicinity of an environment with a large IPC score is expected to display a higher performance in that environment in comparison to its mean performance, and conversely for genotypes located far from that environment on the biplot.

Conclusion

The classical statistical methods used in this study found highly significant genotype x environment interaction, a major challenge confronting cassava breeders in the course of breeding for high yielding and stable varieties. We also observed highly diverse environments, with environment effects accounting for a large percentage of observed variation in the agronomic traits as these traits are polygenic in nature. There were 6 mega-environments identified from 17 testing environments as a function of winning genotypes.

The Finlay-Wilkinson, AMMI, and GGE are fixed effect models and they may not be an appropriate approach to use when estimating quantitative genetic parameters in the presence of unbalanced data and/or when jointly analyzing heterogeneous trial designs. Such circumstances require a mixed model approach where different variance covariance structures can be explored. In addition, these models assumed homogeneity of error variances across the testing environments which may be misleading as error variances were heterogeneous as revealed through likelihood ratio tests. None of these linear bilinear models can account for relatedness among the genotypes, e.g., using relatedness matrices from pedigree and/or molecular data.

Though the same genotypes were evaluated across the testing environments (trials or location by year combination), there were locations (Abuja, Otobi, Umudike, and Ubiaja) where this study was carried out in just one out of three cropping seasons. This caused unbalancedness in the data structure. Therefore, the outcome of delineating the testing environments into mega-environments may be misleading. In future studies, it may be advisable to have more than one cropping season of data from such locations to ensure having well-defined mega environments. To get a clearer picture of locations that are representative of the TPE, future studies may require

many years of historical data. To better understand the factors influencing the GEI, it is advisable to explicitly exploit soil and weather data.

Acknowledgements

The authors thank the UK's Foreign, Commonwealth & Development Office (FCDO) and the Bill & Melinda Gates Foundation (Grant INV-007637, http://www.gatesfoundation.org) for their financial support. We also extend our appreciation to the dedicated staff of the International Institute of Tropical Agriculture (IITA) of the cassava breeding program that assisted with the fieldwork, screen house evaluation and laboratory analyses.

References

1. Egesi CN, Ilona P, Ogbe FO, Akoroda M, Dixon A. Genetic variation and genotype X environment interaction for yield and other agronomic traits in cassava in Nigeria. Agron J. 2007;99(4):1137–42.

2. Tumuhimbise R, Melis R, Shanahan P, Kawuki R. Genotype × environment interaction effects on early fresh storage root yield and related traits in cassava. Crop J. 2014;2(5):329–37.

3. Kvitschal MV, Filho PSV, Scapim CA, Gonçalves-Vidigal MC, Sagrilo E, Pequeno MG, Rimoldi F. Comparison of methods for phenotypic stability analysis of cassava (Manihot esculenta crantz) genotypes for yield and storage root dry matter content. Braz Arch Biol Technol. 2009;52(1):163–75.

4. Kawano K. Thirty years of cassava breeding for productivity - Biological and social factors for success. Crop Sci. 2003;43(4):1325–35.

5. Sayre R, Beeching JR, Cahoon EB, Egesi C, Fauquet C, Fellman J, Fregene M, Gruissem W, Mallowa S, Manary M, Maziya-Dixon B, Mbanaso A, Schachtman DP, Siritunga D, Taylor N, Vanderschuren H, Zhang P. The BioCassava Plus Program: Biofortification of Cassava for Sub-Saharan Africa. Annu Rev Plant Biol. 2011;62(1):251–72.

6. Ceballos H, Kulakow P, Hershey C. Cassava Breeding: Current Status, Bottlenecks and the Potential of Biotechnology Tools. Trop Plant Biol. 2012;5(1):73–87.

7. Kvitschal MV, Vidigal Filho PS, Scapim CA, Gonçalves-Vidigal MC, Pequeno MG, Sagrilo E, Rimoldi F. Evaluation of phenotypic stability of cassava clones by AMMI analysis in northwestern Paraná state. Crop Breed Appl Biotechnol. 2006;6(3):236–41.

8. Gregorius HR, Namkoong G. Joint analysis of genotypic and environmental effects. Theor Appl Genet. 1986;72(3):413–22.

9. Crossa J. From Genotype × Environment Interaction to Gene × Environment Interaction. Curr Genomics. 2012;13(3):225–44.

10. Crossa J, Cornelius PL. Sites regression and shifted multiplicative model clustering of cultivar trial sites under heterogeneity of error variances. Crop Sci. 1997;37(2):406–15.

11. Gauch HG, Zobel RW. Identifying mega-environments and targeting genotypes. Crop Sci. 1997;37(2):311–26.

12. Gauch HGJr. Model Selection and Validation for Yield Trials with Interaction. Published by : International Biometric Society Stable URL : http://www.jstor.org/stable/2531585. 2016;44(3):705–15.

13. Gabriel. The biplot graphic display of matrices with application to principal component analysis. Biometrika. 1971;58:453–67.

14. Kempton RA. The use of biplots in interpreting variety by environment interactions. J Agric Sci. 1984;103(1):123–35.

15. Hagos HG, Abay F. AMMI and GGE biplot analysis of bread wheat genotypes in the northern part of Ethiopia. J Plant Breed Genet. 2013;1(1):12–8.

16. Purchase J. Parametric analysis to describe genotype by environment interaction and yield stability in winter wheat. PhD Thesis. 1997;83–83.

17. Mahmodi N, Yaghotipoor A, Farshadfar E. AMMI stability value and simultaneous estimation of yield and yield stability in bread wheat ('Triticum aestivum'L.). Aust J Crop Sci. 2011;5(13):1837–44.

18. Yan W, Hunt LA, Sheng Q, Szlavnics Z. Cultivar evaluation and mega-environment investigation based on the GGE biplot. Crop Sci. 2000;40(3):597–605.

19. Wickham H. ggplot2: Elegant Graphics for Data Analysis. [Internet]. New York: Springer-Verlag; 2016. Available from: https://ggplot2.tidyverse.org

20. R Core Team. R: A language and environment for statistical computing. R Foundation for Statistical Computing, Vienna, Austria. 2018;

21. SAS Institute Inc. SAS/STAT(®) 13.3 User's Guide. Cary, NC: SAS Institute Inc.; 2013.

22. Mrode RA. Linear Models for the prediction of animal breeding values,. Third ed. CABI, Oxfordshire, UK.; 2014.

23. Bart-Jan van R. statgenGxE: Genotype by Environment (GxE) Analysis. R package version 1.0.4. 2021; Available from: https://cran.r-project.org/package=statgenGxE

24. Finlay KW Wilkinson, GN. THE ANALYSIS OF ADAPTATION IN A PLANT-BREEDING PROGRAMME and G. N. WILKINSON? By K. W. FINLAY*. Aust J Agric Res. 1963;14(1958):742–54.

25. Malosetti M, Ribaut JM, van Eeuwijk FA. The statistical analysis of multi-environment data: Modeling genotype-by-environment interaction and its genetic basis. Front Physiol. 2013;4 MAR(March):1–17.

26. Falcon CM, Kaeppler SM, Spalding EP, Miller ND, Haase N, AlKhalifah N, Bohn M, Buckler ES, Campbell DA, Ciampitti I. Relative utility of agronomic, phenological, and morphological traits for assessing genotype- by- environment interaction in maize inbreds. Crop Sci. 2020;60(1):62–81.

27. Gauch HGJr. Model Selection and Validation for Yield Trials with Interaction. Biometrics. 1988;44(3):705–15.

28. Farshadfar E, Sutka J. Biplot analysis of genotype-environment interaction in durum wheat using the ammi model. Acta Agron Hung. 2006;54(4):459–67.

29. Yan W, Kang MS, Ma B, Woods S, Cornelius PL. GGE biplot vs. AMMI analysis of genotype-by-environment data. Crop Sci. 2007;47(2):643–55.

30. Hongyu K, Silva F, Oliveira A, Sarti DA, Araújo L, Dias C. Comparação entre os modelos AMMI e GGE-Biplot para os dados de ensaios multi-ambientais. Rev Bras Biom. 2015;33(2):139–55.

31. Yan W, Kang MS. GGE biplot analysis: A graphical tool for breeders, geneticists, and agronomists. CRC Press, Boca Raton, FL.; 2003.

32. Yan W. GGE biplot vs. AMMI graphs for genotype-by-environment data analysis. J Indian Soc Agric Stat. 2011;65(2):181–93.

33. Lin C-S, Binns MR. A superiority measure of cultivar performance for cultivar× location data. Can J Plant Sci. 1988;68(1):193–8.

34. Cullis BR, Smith AB, Coombes NE. On the design of early generation variety trials with correlated data. J Agric Biol Environ Stat. 2006;11(4):381–93.

35. Ward JH. Hierarchical Grouping to Optimize an Objective Function. J Am Stat Assoc. 1963;58(301):236–44.

36. Dyke G, Lane P, Jenkyn J. Sensitivity (stability) analysis of multiple variety trials, with special reference to data expressed as proportions or percentages. Exp Agric. 1995;31(1):75–87.

37. Jiwuba L, Danquah A, Asante I, Blay E, Onyeka J, Danquah E, Egesi C. Genotype by Environment Interaction on Resistance to Cassava Green Mite Associated Traits and Effects on Yield Performance of Cassava Genotypes in Nigeria. Front Plant Sci. 2020;11:572200.

38. Benesi I, Labuschagne M, Dixon A, Mahungu N. Genotype x enviroment interaction effects on native cassava starch quality and potential for starch use in the commercial sector. Afr Crop Sci J. 2004;12(3):205–16.

39. Nduwumuremyi A, Melis R, Shanahan P, Theodore A. Interaction of genotype and environment effects on important traits of cassava (Manihot esculenta Crantz). Crop J. 2017;5(5):373–86.

Supporting information

S1 Fig. This is the scatter plot of coefficient of variation and heritability.

S2 Fig. This is the scatter plot of experimental accuracy and heritability.

S3 Fig. This is the boxplot showing distribution of observed traits for some parameter estimates.

S4 Fig. This is the scatter plot of cultivar superiority index and mean fresh root yield.

S5 Fig. This is the scatter plot of cultivar superiority index and mean dry matter content.

S6 Fig. This is the scatter plot of cultivar superiority index and mean dry yield.

S7 Fig. This is clustering of environments based on genotypic blups of fresh root yield.

S8 Fig. This is clustering of environments based on genotypic blups of dry matter content.

S9 Fig. This is clustering of environments based on genotypic blups of top yield.

S1 Table. This is the summary statistics of individual trials

S2 Table. This is the likelihood ratio test of absence versus presence of GEI

S3 Table. This is the likelihood ratio test of homogeneity versus heterogeneity of error variances.

S4 Table. This is the ANOVA table showing the variance component estimates.

S5 Table. This is ANOVA table showing the partition of GEI variance component.

S6 Table. This is the ANOVA table resulting from Finlay-Wilkinson (FW) model.

S7 Table. This is the genotypes ranking based on sensitivities values from FW model.

S8 Table. This is minimum, median, maximum, and variance of MSE from FW model.

S9 Table. This is minimum, median, maximum, and variance of slopes from FW model.

S10 Table. This is the combined ANOVA table resulting from AMMI model.

S11 Table. This is the ranking of genotypes based on AMMI stability value and genotype selection index.

S12 Table. This is the table showing mega-environment based on AMMI2 model.

S13Table. This is the combined ANOVA table resulting from GGE model.

S14 Table. This is the assessment of stability of genotypes based on cultivar superiority index.

S15 Table. This is the ranking of testing environments for dry matter content based on correlation and Cullis heritability.

S16 Table. This is the ranking of testing environments for top yield based on correlation and Cullis heritability.

Click here to access/download;Figure;Fig1.tiff

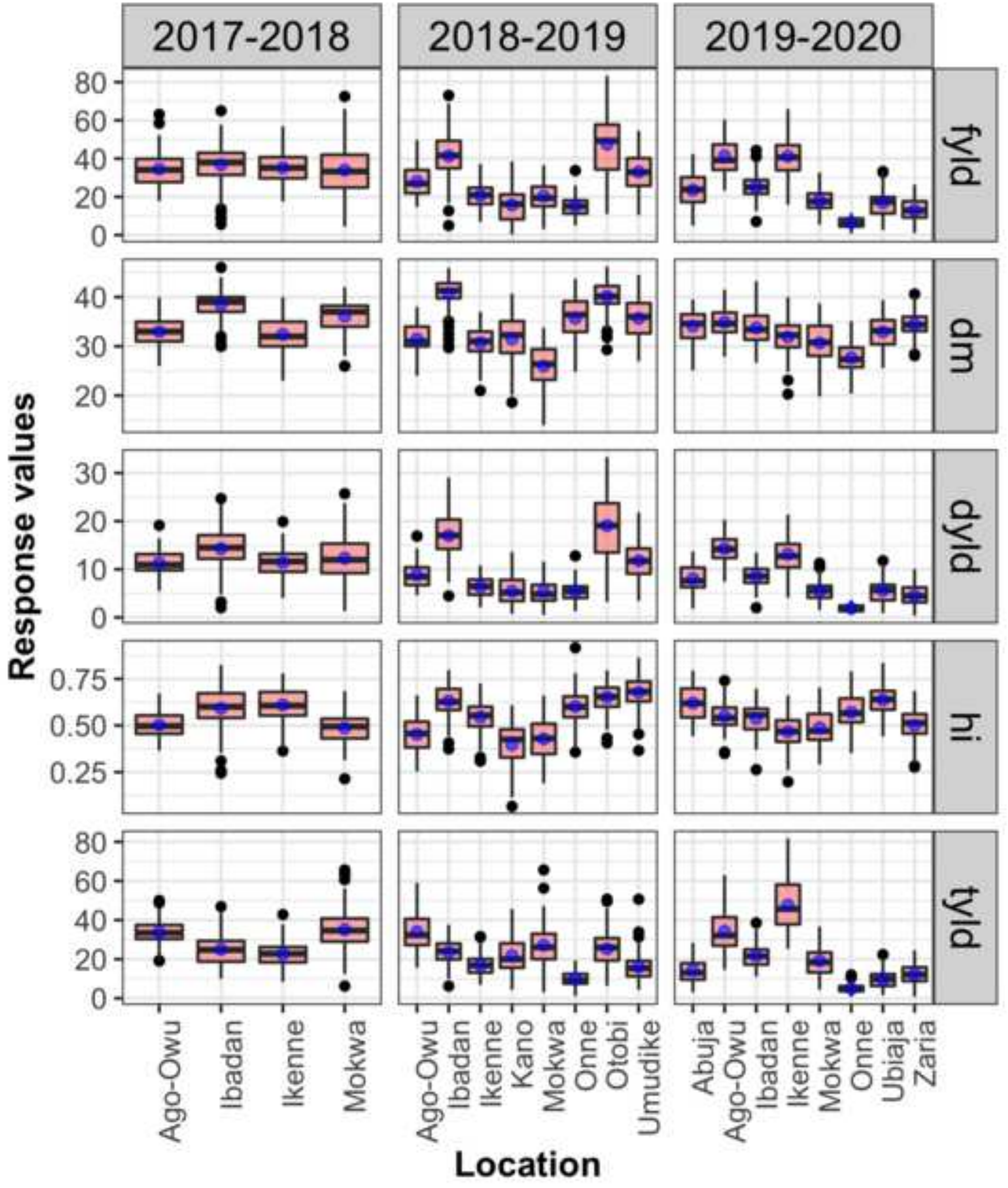

Click here to access/download;Figure;Fig2.tiff

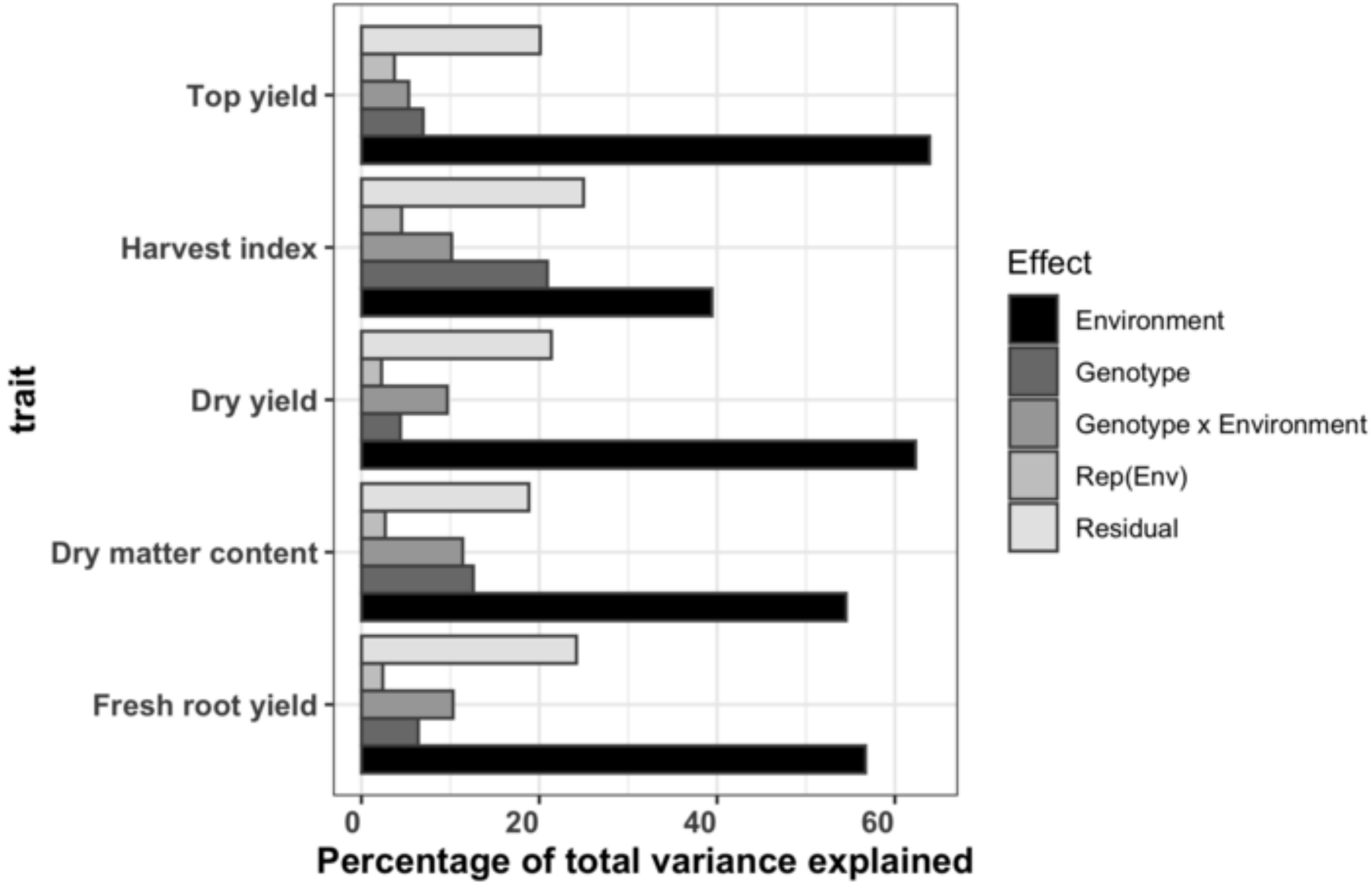

Figure

Click here to access/download;Figure;Fig3.tiff

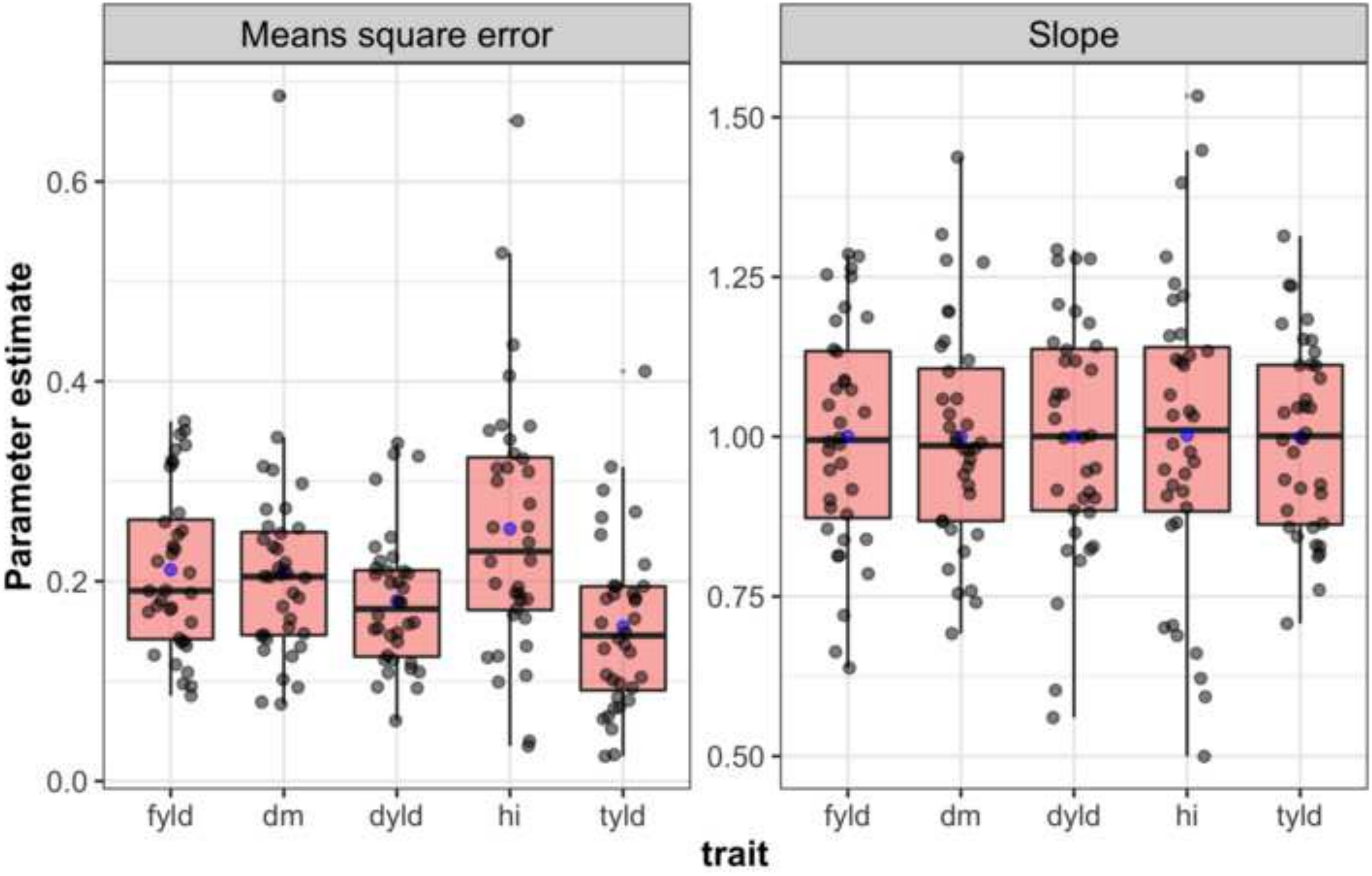

Figure
Click here to access/download;Figure;Fig4.tiff

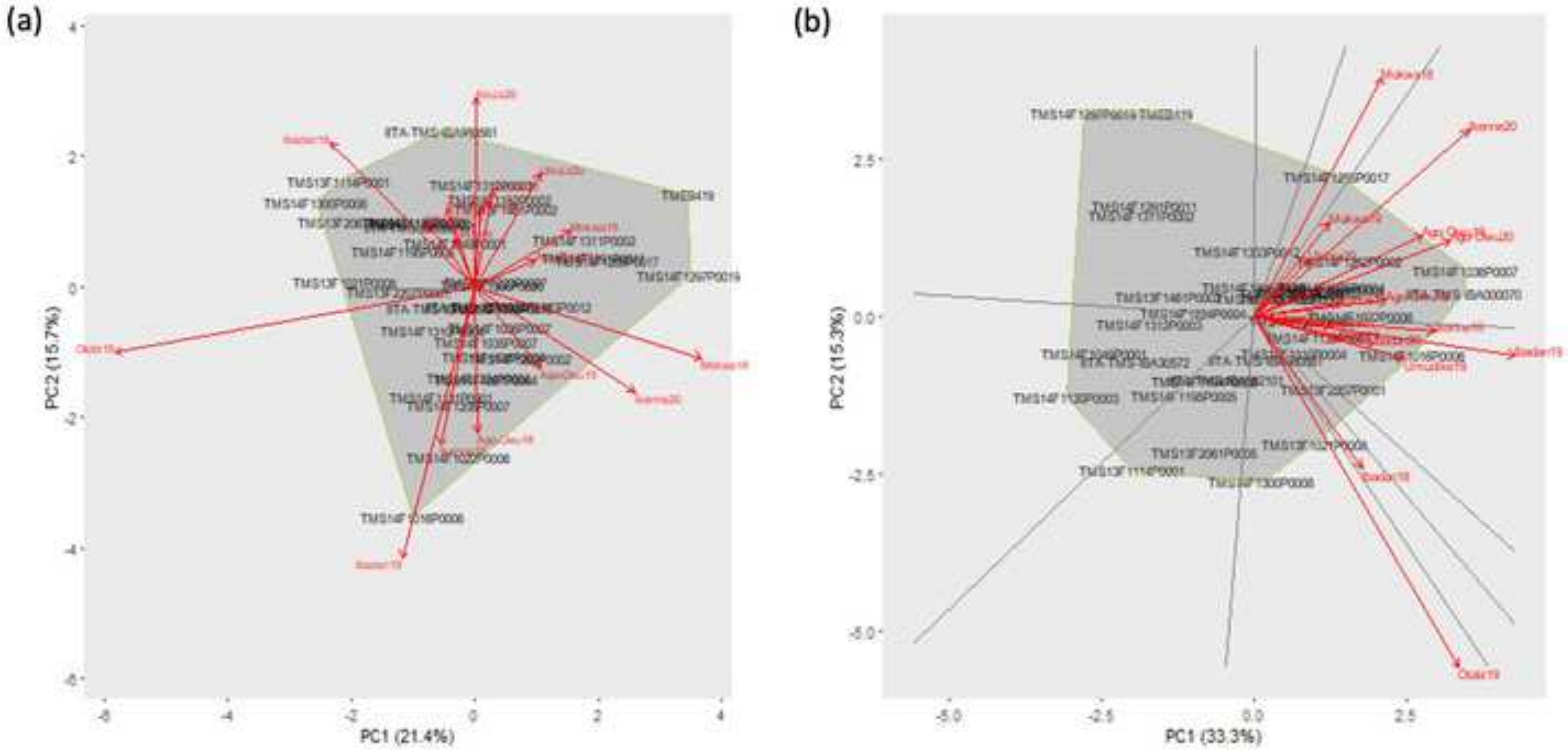

Figure

Click here to access/download;Figure;Fig5.tiff

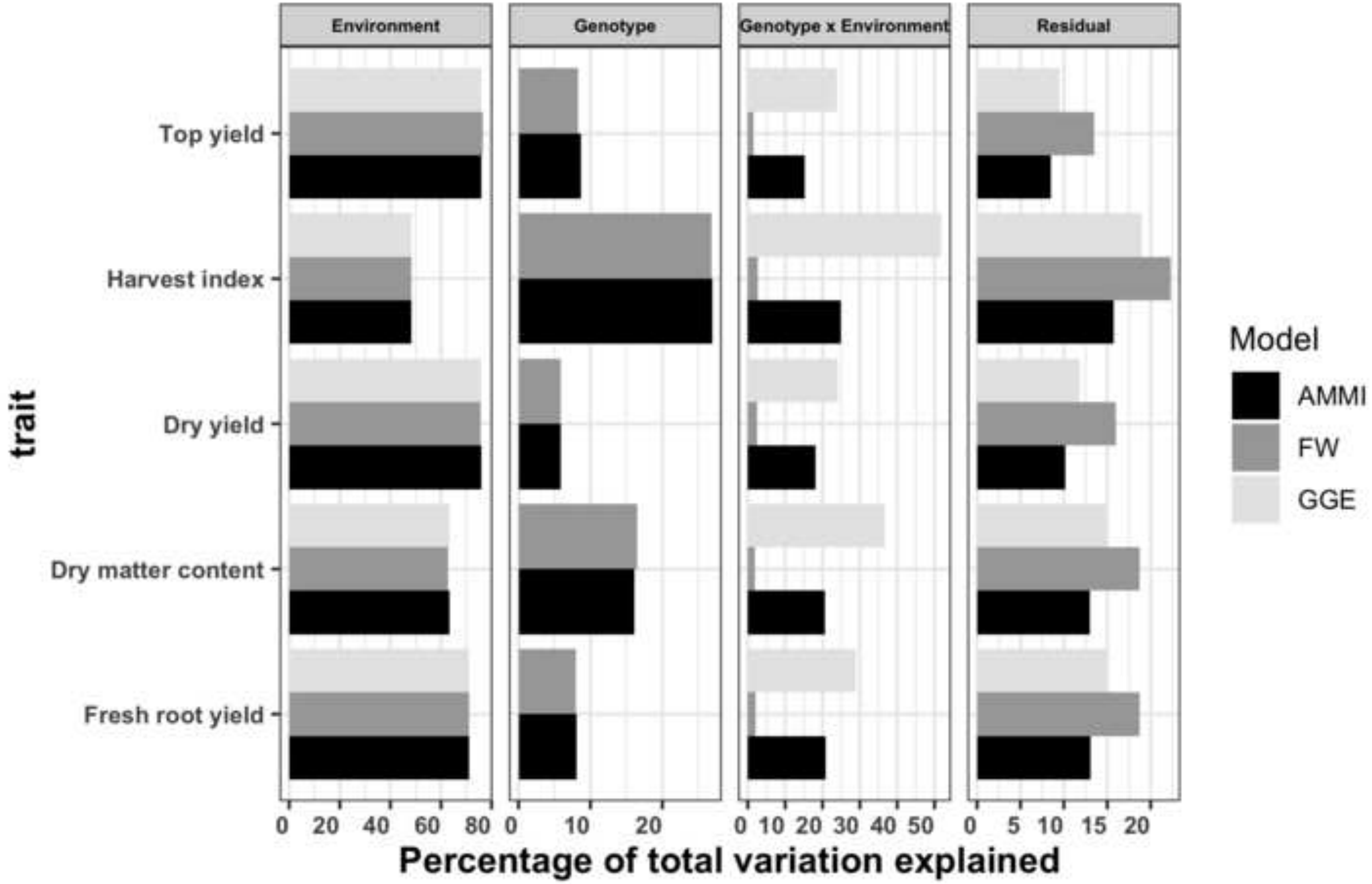

Click here to access/download;Figure;Fig6.tiff

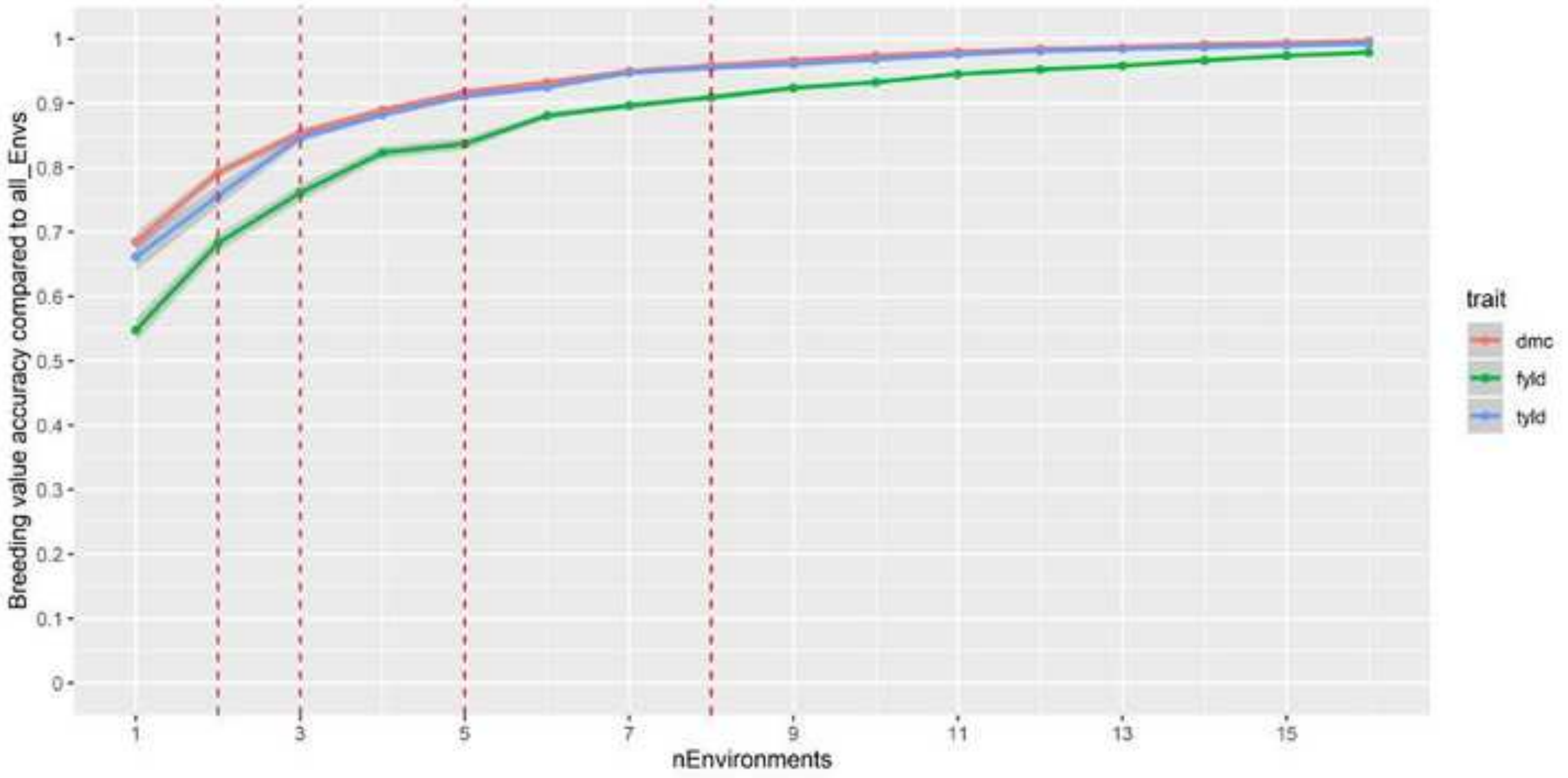

Click here to access/download
Supporting Information - Compressed/ZIP File Archive
supporting_information.zip

Click here to download Data Review URL
https://github.com/mab658/classical_analysis_GxE
