# Supplementary material for: Exploring genotype by environment interaction on cassava yield and yield related traits using classical statistical methods
Source: PLoS One. 2022 Jul 18;17(7):e0268189. doi: 10.1371/journal.pone.0268189 (PMC9292083; doi:10.1371/journal.pone.0268189)
Supplement: Attachment — Submitted filename: Review PONE-D-21-40030.docx [file pone.0268189.s026.docx]

I have reviewed the manuscript # PONE-D-21-40030,

Title “Exploring genotype by environment interaction on cassava yield and yield related traits using classical statistical methods”.

I am glad to express that the manuscript is well written, and the message is clearly presented. The standard of language is also good. The introduction is good and clearly describes the background of the study. The analytical methods used, and the conclusions drawn are satisfactory.

Although the research seems more of regional importance rather than international, but I think it’s still good enough to contribute to the global knowledge. The statistical methods also look conventional. But keeping in mind the current situation of lack of facilities for advanced genomics tools for every researcher, the guidelines provided for conventional methods will be of valuable importance to make genetic gains on a very important problem of GxE in cassava.

Having said that, I still want to suggest some improvements to improve the manuscript, such as

1. There are a few minor typo errors, I have mentioned them as sticky notes on the attached pdf document.
2. In materials and methods section, please add some salient features/background information of the clones/genotypes used in the study, to indicate why they were selected for the experiment. The relevant information can be added to Table 1, the table also needs improvement in formatting.
3. In materials and methods section, it is suggested that instead of showing coordinates of study sites in tabular form (Table 2) they can be displayed in a form of a map with study sites displayed as dots.
4. Similarly in materials and methods section, the weather data in Table 2 can be displayed in a graphical form for a clear understanding of readers.
5. The experimental material is designated with 2 different terms in various section of the manuscript such as
   a. “clones” in abstract and material & methods sections
   b. “genotypes” in results & discussion sections. It should be consistent throughout
6. I suggest that conclusion should be reconsidered so that it can show that based on the results all the objectives of the study were achieved.

To summarise it, I recommend that the manuscript may be considered suitable for publication with minor revisions.
